# Construction of a high density genetic linkage map to define the locus conferring seedlessness from Mukaku Kishu mandarin

**DOI:** 10.3389/fpls.2023.1087023

**Published:** 2023-02-14

**Authors:** Krishan Kumar, Qibin Yu, Dharminder Bhatia, Chitose Honsho, Frederick G. Gmitter

**Affiliations:** ^1^ Citrus Research and Education Center, Institute of Food and Agricultural Sciences, University of Florida, Lake Alfred, FL, United States; ^2^ Punjab Agricultural University, Dr. JC Bakhshi Regional Research Station, Abohar, India; ^3^ Department of Plant Breeding and Genetics, Punjab Agricultural University, Ludhiana, India; ^4^ Laboratory of Pomology, Faculty of Agriculture, University of Miyazaki, Miyazaki, Japan

**Keywords:** mandarin, consensus map, Mukaku Kishu, seedlessness, SNP array

## Abstract

Mukaku Kishu (‘MK’), a small sized mandarin, is an important source of seedlessness in citrus breeding. Identification and mapping the gene(s) governing ‘MK’ seedlessness will expedite seedless cultivar development. In this study, two ‘MK’-derived mapping populations- LB8-9 Sugar Belle^®^ (‘SB’) × ‘MK’ (N=97) and Daisy (‘D’) × ‘MK’ (N=68) were genotyped using an *Axiom_Citrus56 Array* encompassing 58,433 SNP probe sets, and population specific male and female parent linkage maps were constructed. The parental maps of each population were integrated to produce sub-composite maps, which were further merged to develop a consensus linkage map. All the parental maps (except ‘MK_D’) had nine major linkage groups, and contained 930 (‘SB’), 810 (‘MK_SB’), 776 (‘D’) and 707 (‘MK_D’) SNPs. The linkage maps displayed 96.9 (‘MK_D’) to 98.5% (‘SB’) chromosomal synteny with the reference Clementine genome. The consensus map was comprised of 2588 markers including a phenotypic seedless (*Fs*)-locus and spanned a genetic distance of 1406.84 cM, with an average marker distance of 0.54 cM, which is substantially lower than the reference Clementine map. For the phenotypic *Fs*-locus, the distribution of seedy and seedless progenies in both ‘SB’ × ‘MK’ (55:42, χ^2^ = 1.74) and ‘D’ × ‘MK’ populations (33:35, χ^2^ = 0.06) followed a test cross pattern. The *Fs*-locus mapped on chromosome 5 with SNP marker ‘AX-160417325’ at 7.4 cM in ‘MK_SB’ map and between two SNP markers ‘AX-160536283’ and ‘AX-160906995’ at a distance of 2.4 and 4.9 cM, respectively in ‘MK_D’ map. The SNPs ‘AX-160417325’ and ‘AX-160536283’ correctly predicted seedlessness of 25-91.9% progenies in this study. Based on the alignment of flanking SNP markers to the Clementine reference genome, the candidate gene for seedlessness hovered in a ~ 6.0 Mb region between 3.97 Mb (AX-160906995) to 10.00 Mb (AX-160536283). This region has 131 genes of which 13 genes (belonging to seven gene families) reportedly express in seed coat or developing embryo. The findings of the study will prove helpful in directing future research for fine mapping this region and eventually underpinning the exact causative gene governing seedlessness in ‘MK’.

## Introduction

Edible citrus comprises a group of fruits mainly mandarins, sweet oranges, lime, lemons, pummelos, and grapefruit. These fruits are valued for their nutritive and health promoting abilities. Among the various citrus types, mandarins have primary utility as a fresh fruit. The major breeding objectives for mandarins are high eating quality, seedlessness, easy peelability, and round the year availability of fruit (Navarro et al., 2015). Conventional hybridization has been the most important method for genetic improvement of mandarins, but it is costly as well as challenging. For example, the release of LB8-9 Sugar Belle^®^ (‘SB’) took 24 years from the year of its original cross. The integration of the molecular markers into the hybridization-based breeding program can expedite the pace of varietal development in citrus (Gmitter et al., 2007). The use of closely associated markers can allow selection of the desirable progenies many years before the evaluation for the targeted trait becomes possible, thus, help in compressing the breeding cycle (Dirlewanger et al., 2004).

Successful employment of marker assisted selection requires linkage maps with wide genomic coverage. The availability of genome sequences of different citrus cultivars and accessions has helped to improve the resolution of linkage maps through the informative expressed sequences (EST) derived EST-SSRs (Chen et al., 2008), gene-derived cleaved amplified polymorphic sequences (CAPS) (Shimada et al., 2014) and single nucleotide polymorphism based markers (SNPs) (Ollitrault et al., 2012; Chen and Gmitter, 2013; Yu et al., 2016). SNPs are the most abundant DNA markers which are evenly distributed on a whole genome and can tag almost any gene or locus of a genome (Brookes, 1999). With rapid developments in next generation sequencing technologies and availability of reference whole genome sequences, SNPs have become the marker of choice in genetics studies. SNP array-based genotyping platforms have been considered useful for developing high density linkage maps, gene/QTL mapping, and marker-assisted crop breeding. Their use over multiple populations also provides opportunity for development of integrated linkage maps for higher resolution of the loci conferring the target trait (Cui et al., 2017). High throughput markers such as DArTseq markers (Curtolo et al., 2017; Curtolo et al., 2018) and SNP markers through genotype by sequencing (GBS) technology (Huang et al., 2018) or by SNP array platforms (Yu et al., 2016) have been used to develop high density linkage maps in citrus. Previously, a medium density 1536 Illumina Golden Gate SNP-array was used to construct a mandarin linkage map (Yu et al., 2016), but a more dense *Axiom*_*Citrus56 Array* encompassing of 58,433 SNP probe sets became available for genotyping in citrus (Hiraoka, 2020).

Seedlessness is required for new mandarin cultivars by the marketplace. Citrus breeders have used Mukaku Kishu (‘MK’) as pollen parent in crosses with seedy monoembryonic diploid parents to produce seedless varieties (Nesumi et al., 2001). Seedlessness can be achieved through interploid hybridization in citrus (Grosser and Gmitter, 2005; Aleza et al., 2012a; Aleza et al., 2012b), or by exploiting cytoplasmic male sterility (CMS) of the Satsuma group of mandarins (Goto et al., 2018) or from self-incompatible pollen-pistil interaction (Montalt et al., 2021). However, triploid breeding requires the synthesis of tetraploids or exploitation of unreduced gametes, and recovery of triploid embryos generally is accomplished through embryo culture technique (Aleza et al., 2012a); further, some triploid hybrids can have occasionally very few seeds in their fruit, particularly with cross pollination. Similarly, induction of seedlessness using the CMS system is dependent on the cooperative action of the cytoplasmic and nuclear genes derived from Kishu and Satsuma mandarin (Yamamoto et al., 1997; Goto et al., 2018). On the other hand, self-incompatibility produces seedless fruits in self-pollination events. The response of self-incompatibility can also be influenced by environment (Aloisi et al., 2020). In contrast to these approaches, the seedlessness of ‘MK’ is governed by nuclear genes (Nesumi et al., 2001). The complete seedlessness in ‘MK’ is caused by developmental arrest of fertilized seeds. It is hypothesized that a two gene system possibly determines the seedlessness of ‘MK’: *Fs* a dominant gene that induces seedlessness, and *Is*, a repressor gene which in dominant state inhibits seedlessness. The allelic constitution of these two genes for ‘MK’ is reported to be ‘*Fsfs-isis*’ and for seedy mandarins as ‘*fsfs-isis*’ (Yamasaki et al., 2007). However, Nesumi et al. (2001) suggested that for mandarin crosses, this type of seedlessness is primarily determined by a single major locus. Several mandarin populations involving ‘MK’ as pollen parent have been developed, and high-resolution linkage maps can provide the exact location of genes controlling various traits as well as the markers closely associated with them. Previous attempts to identify and map genes governing seedlessness from ‘MK’ used RAPD markers or some limited number of gene-based markers (Shimada et al., 2014), but those identified were family-specific (Chavez and Chaparro, 2011). In this study, high density linkage maps for ‘SB’, Daisy (‘D’), ‘MK’ were constructed by genotyping with *Axiom_Citrus*56 *SNP Array*, these maps were integrated to create a consensus mandarin map, which was used to identify and map the locus governing the seedlessness trait in ‘MK’.

## Materials and methods

### Mapping populations

**Figure 1:**
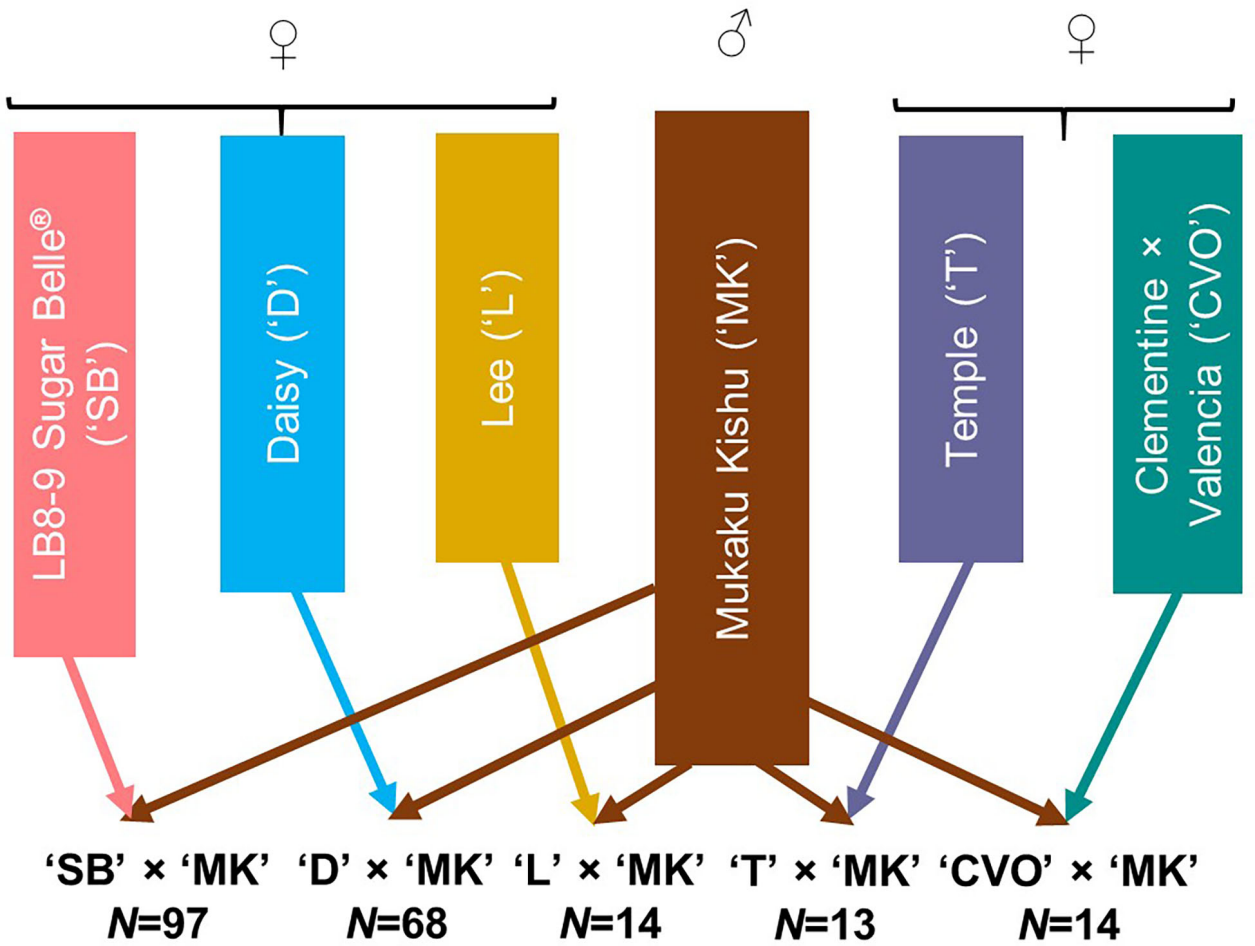
Description of the five F_1_ mandarin populations used for genotyping with *Axiom_Citrus56AX array. N* represents the number of individuals in each population.

The 206 progeny individuals from five different F_1_ mandarin populations growing at the University of Florida-IFAS Citrus Research and Education Center (Lake Alfred, FL) were used in this study (**Figure 1**). Mukaku Kishu (*Citrus reticulata* Blanco) (‘MK’), a completely seedless mandarin cultivar, was the common male parent in all the populations. All five maternal mandarin parents, ‘SB’ (Clementine mandarin × Minneola tangelo), ‘D’ [(Clementine mandarin × Orlando tangelo) × (Clementine mandarin × Ponkan mandarin)], Temple (‘T’) (a natural mandarin × sweet orange hybrid), Lee (‘L’) (Clementine mandarin × Orlando tangelo), and Clementine × Valencia orange (‘CVO’) produce fruit containing monoembryonic seeds. All of these, except ‘CVO’ are released commercial cultivars. To preclude any inadvertent inclusion of off types/nucellars in the mapping population, the hybridity of the population individuals (for ‘MK’ paternity) was verified through few homozygous SNPs polymorphic between the maternal parents and ‘MK’. The individuals with doubtful identity were not used in this study. All the populations were fruiting in the 2017-18 season.

### Phenotyping of progeny for seed content

The fruits of the 206 F_1_ progenies were examined for presence or absence of seeds in December 2017. For each progeny, ten fruits were cut transversely into two halves, juice was squeezed, and the fruits were observed for the seeds. Progenies containing even a single seed in the fruit were scored as seedy, while those without seed as seedless.

### Genotyping with *Axiom_Citrus56 Array* and data filtering

The genomic DNA of the parents and the progenies was isolated using *Qiagen DNeasy Plant Minikit* and quantified using NanoDrop™. The samples were prepared and outsourced to Thermo Fisher Scientific Inc. for genotyping with *Axiom_Citrus56 Array* encompassing 58,433 SNP probe sets. The genotyping data were filtered through Axiom Analysis Suite 5.1.1 software (Thermo Fisher Scientific Inc, 2020) by selecting *Axiom_Citrus56.r1* array and *Best Practices Workflow.* The sample QC (Dish quality control: ≥ 0.82, QC call rate: ≥ 0.97, Percent of passing samples: ≥ 95, Average call rate for passing samples: ≥ 98.5) and SNP QC were as per the default configurations of *Diploid.legacy.v5* under the *Threshold settings*. The data were processed together for the whole set of 210 samples [206 progenies and four parents namely ‘SB’, ‘D’, ‘T’ and ‘MK’)]; as well as separately for two major populations *i.e.* ‘SB’ × ‘MK’ (N=97) and ‘D’ × ‘MK’ (N=68). All three analyses were performed using *default* analysis configuration of *Axiom_Citrus56_96orMore.r1*, the only option available for *Axiom_Citrus56.r1* array in the software. The SNP probe sets were classified into six classes based on the properties of cluster formation: i. Poly High Resolution (PHR) - three highly resolved clusters (two homozygous and one heterozygous); ii. No Minor Homozygote (NMH) - two highly resolved clusters (one homozygous and one heterozygous); iii. Mono High Resolution (MHR) - only one homozygous cluster; iv. Off Target Variant (OTV) - three well resolved clusters with an additional off target cluster; v. Call Rate Below Threshold (CRBT) - Call rate was below threshold (0.97) but other cluster properties were above the threshold; and vi. Others- SNPs not grouped in any of the previous categories. The analyzed results were exported as text file.

### Genotype coding and construction of parental linkage maps

The progenies of ‘SB’ × ‘MK’ and ‘D’ × ‘MK’ populations were used for linkage map construction using JoinMap version 4.1 (Van Ooijen, 2006; Van Ooijen, 2011). For each population, the SNPs exhibiting 1:1 (heterozygous × homozygous for female parent, homozygous × heterozygous for male parent) and 1:2:1 segregation pattern (heterozygous for both parents) were used for genetic mapping. Depending upon the parental segregation direction, the original calls of the SNPs were substituted with the codes of *‘nn’* and *‘np’* (for SNPs segregating from male parent) and *‘lm’* and ‘*ll’* (for SNPs segregating from female parent). The co-segregating (1:2:1) SNPs were coded as *‘hh’*, *‘hk’* or *‘kk’*, depending upon their genotype. The linkage analyses and maps were constructed using cross pollination (CP) model in JoinMap 4.1 following two way pseudo test cross approach that allows generation of separate maps for male and female parents (Grattapaglia and Sederoff, 1994). Before linkage analysis, the following classes of SNPs were sequentially removed: SNPs with missing data for > 10% of the progenies; exhibiting 100% similarity in segregation pattern with another locus in the dataset; or having segregation pattern significantly skewed from 1:1 or 1:2:1 Mendelian ratio (*P<* 0.005). Further, the loci showing 99% similarity were also eliminated. In each population, initially, the *‘nn × np’* and *‘lm × ll’* datasets were used for developing male and female specific linkage maps. The selected ‘*hk* × *hk’* markers were then combined with male and female parent specific datasets to reconstruct male and female linkage maps. The linkage grouping was performed with grouping static independence LOD that permits use of markers with distorted segregation without inducing spurious linkage (Bernet et al., 2010; Ollitrault et al., 2012; Huang et al., 2018). The linkage groups were obtained at independence LOD threshold of 6.0 and recombination fraction of 0.4. The linkage groups were numbered according to the chromosomal ID of the SNPs in *Axiom_Citrus56 Array*. The SNP markers were ordered using regression mapping algorithm while map distance (cM) was calculated using Kosambi mapping function. All the linkage maps were drawn with MapChart 2.32 (Voorrips, 2002).

### Construction of integrated ‘MK’ map and consensus linkage map

Since both the mapping populations were constructed using the common pollen parent ‘MK’, it enabled the development of an integrated ‘MK’ linkage map. The homologous linkage groups from individual ‘MK’ maps- ‘MK_SB’ (derived from ‘SB’ × ‘MK’ population) and ‘MK_D’ (derived from ‘D’ × ‘MK’ population) were selected and grouped using *‘Combine Groups for Map Integration’* function in the JoinMap 4.1. The consensus linkage map was prepared in two steps. In the first step, the homologous male and female parent specific linkage groups were selected based on the sharing ‘*hk’* markers in each population. The homologues were combined to make sub-composite linkage maps using the function *‘Combine Groups for Map Integration’* in the JoinMap. In the 2^nd^ step, the homologous sub-composite linkage groups from two populations were combined to generate consensus linkage map using online Merge Map software (Wu et al., 2008; Peng et al., 2016) (http://www.mergemap.org/).

### Evaluation of the selected SNPs to identify the seedless progenies

From the mapping analysis, seedless locus linked SNPs were identified. These SNPs were assessed for predicting the seedless progenies in different populations. For this purpose, the actual allelic calls of these SNPs were observed in the progenies and the allelic pattern associated with seedlessness was identified. The marker ability to identify the true seedless progenies was determined from its positive prediction value (PPV) for seedless progeny detection.


$$\text{PPV for seedless progeny detection (\%) = }\frac{\begin{array}{c} \text{Actual seedless progenies among the predicted}\\ \text{progenies} \end{array}}{\begin{array}{c} \text{Total seedless progenies predicted by the }\\ \text{SNP } \end{array}}\times 100$$

### Prediction of candidate genes for seedlessness

The SNP markers flanking the seedless locus were identified on the individual ‘MK’ maps- ‘MK_SB’ and ‘MK_D’. These SNPs were aligned to the annotated Clementine reference genome and the physical interval for the seedless locus was delineated. The total number of genes in these intervals was identified and their function was explored in the model plant *Arabidopsis* and other crops.

## Results

### Fruit phenotyping for seedlessness

**Figure 2:**
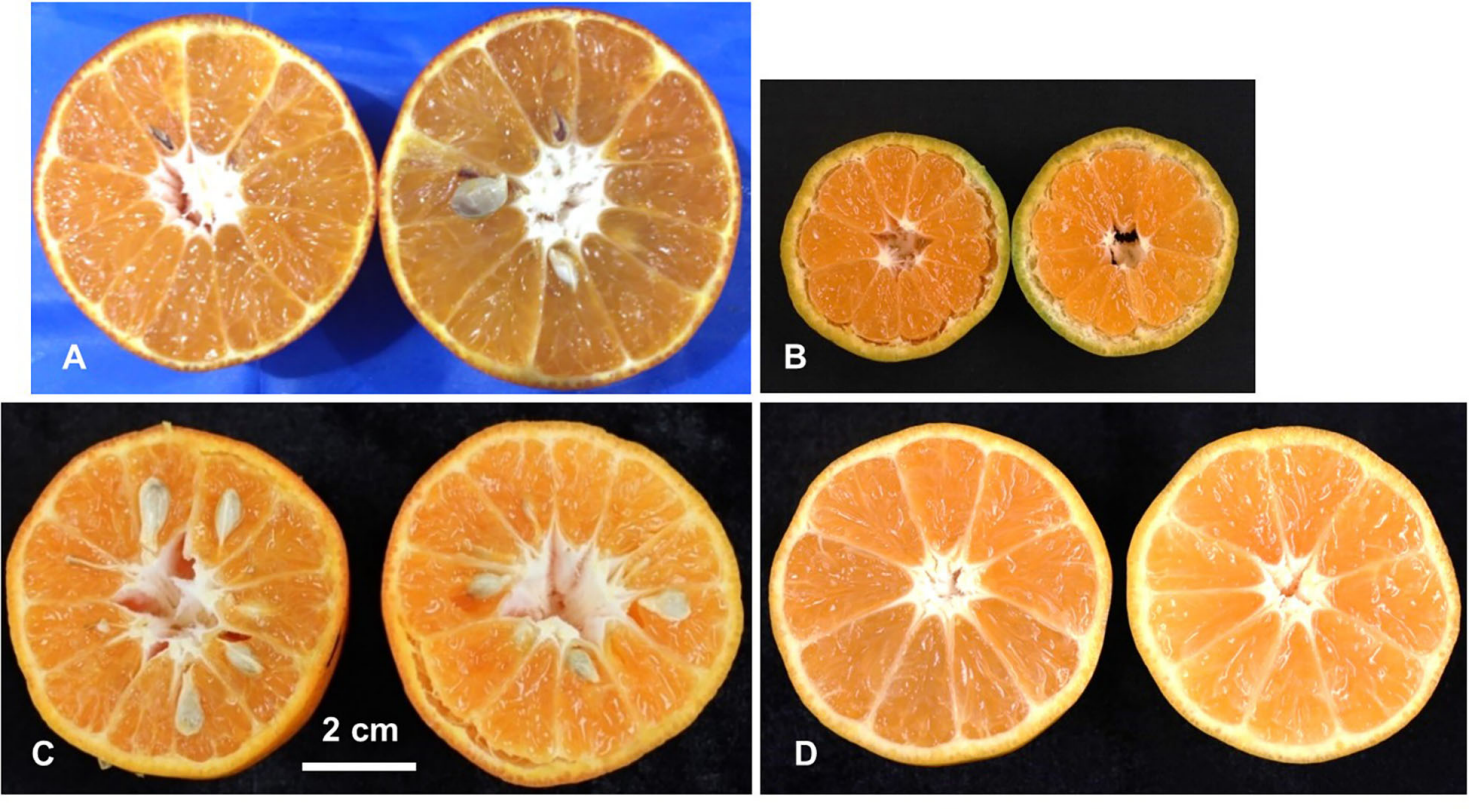
Phenotyping of the parents and population for presence/absence of seeds. Female parent LB8-9 Sugar Belle^®^ (‘SB’) **(A)**, male parent Mukaku Kishu (‘MK’) **(B)**, and their derived F_1_ progenies with seedy **(C)**, and seedless **(D)** phenotypes. Scale bar denotes 2 cm size.

For seeds in fruits, the progeny individuals could be classified into two categories: seedy (fully formed seeds), seedless (no seeds) (**Figure 2**). In the two major populations, the ratio of seeded to seedless was 55:42 (χ^2^ = 1.74) in ‘SB’ × ‘MK’ population and 33:35 (χ^2^ = 0.06) in ‘D’ × ‘MK’ populations, which fit the test cross distribution. These observations showed agreement with the hypothesis of Nesumi et al. (2001) that the seedlessness in crosses of seedy mandarins × ‘MK’ is governed by a major locus (*Fs*).

### SNP genotyping and identification of polymorphic SNPs

Analysis of genotyping data of four parents (‘SB’, ‘D’, ‘T’ and ‘MK’) and 206 progeny individuals with Axiom Analysis Suite software revealed the presence of six types of SNP markers (described under Material and Methods), in variable proportions i.e. MHR: 52.13%, PHR: 22.49%, NMH: 17.71%, CRBT: 1.13%, OTV: 0.40% and Others: 6.14% (**Table 1**). The cumulative share of the polymorphic SNP markers (PHR and NMH) was 40.2% over the whole set of samples. The percentage of polymorphic SNP markers varied from 30.77% in ‘D’ × ‘MK’ population (10,352 NMH, and 7,623 PHR) to 40.1% in ‘SB’ × ‘MK’ population (12,669 NMH, and 10,761 PHR).

**Table 1:** Genotyping results of *Axiom Citrus 56AX array* over all 210 samples and two major mapping populations.

Marker type	Genotyped samples					
	Total 210 samples		‘SB’ × ‘MK’ population		‘D’ × ‘MK’ population	
	Counts	Percentage	Counts	Percentage	Counts	Percentage
Poly High Resolution (PHR)	13138	22.49	10761	18.42	7623	13.05
No Minor Homozygote (NMH)	10350	17.71	12669	21.68	10352	17.72
Mono High Resolution (MHR)	30463	52.13	31682	54.22	36825	63.02
Call Rate Below Threshold (CRBT)	660	1.13	838	1.43	1164	1.99
Off Target Variant (OTV)	235	0.40	149	0.25	185	0.31
Others	3587	6.14	2334	3.99	2284	3.91
Total	58433		58433		58433	

### Processing of polymorphic SNPs

As the seedless locus (hereafter mentioned as *Fs*-locus) behaved as a test cross marker and segregated from ‘MK’, the seeded and seedless progenies were genotyped as *‘nn’* and *‘np’*, respectively. The *Fs*-locus was included in the male parent dataset (‘*nn × np’*) of the two mapping populations for establishment of linkage groups and map generation. From the polymorphic SNP loci, the loci producing calls in both the parents of a population were selected. As a result, a total of 10,037 SNPs (5863 ‘*lm* × *ll’*, 2320 ‘*nn × np’* and 1854 ‘*hk* × *hk’*) for ‘SB’ × ‘MK’ and 11,744 SNPs (6241 ‘*lm* × *ll’*, 2964 ‘*nn* × *np’* and 2539 ‘*hk* × *hk’*) for ‘D’ × ‘MK’ populations were obtained (**Table 2**). After removal of SNPs with > 10% missing genotypes, across loci similarity of ≥ 0.99 and showing segregation distortion from 1:1 or 1:2:1 (*P<* 0.005), a total of 1521 parent specific SNP markers (817 ‘*lm* × *ll’*, 704 ‘*nn* × *np’*) in ‘SB’ × ‘MK’ and 1262 markers (664 ‘*lm* × *ll’*, 598 ‘*nn* × *np’*) in ‘D’ × ‘MK’ populations were left for mapping (**Table 2**). The proportion of loci showing ≥ 99% similarity was 76.9% in ‘D’ × ‘MK’ population and 57.1% in ‘SB’ × ‘MK’ population. The number of markers showing segregation distortion from 1:1 or 1:2:1 Mendelian ratio (*P<* 0.005) was higher in ‘SB’ × ‘MK’ population than that of ‘D’ × ‘MK’ population (**Table 2**). The processed parent specific SNP markers were combined with 114 ‘*hk* × *hk’* type markers (heterozygous in both parents) in ‘SB’ × ‘MK’ and with 112 *‘hk* × *hk’* type markers in ‘D’ × ‘MK’ populations to prepare male and female specific linkage maps. Of the ‘*hk’* markers, 91 were common in both populations (**Supplementary Table 1**). The step-by-step procedure used in selection of the ‘*hk’* markers is described in **Supplementary Information 1**.

**Table 2:** Processing of markers for constructing linkage maps in ‘SB’ × ‘MK’ and ‘D’ × ‘MK’ populations.

Particulars	Segregation pattern of markers							
	‘SB’ × ‘MK’ population				‘D’ × ‘MK’ population			
	*lm × ll*	*nn × np^#^*	*hk × hk*	Total	*lm × ll*	*nn × np*	*hk × hk*	Total
Total loci producing calls in two parents	5863	2320	1854	10037	6241	2964	2539	11744
Markers with > 10% missing genotypes	1189	424	–	1613	292	146	–	438
Markers with ≥ 99% similarity	3744	1130	858	5732	5279	2153	1606	9038
Markers with significantly skewed segregation (*P< 0.005*)	113	62	71	246	6	67	29	102
Total processed markers	817	704	114^$^	1635	664	598	112^$^	1374
After combining heterozygous markers	931	818	–		776	710	–	

^#^The nn × np dataset includes phenotypic Fs-locus for both the populations.

^$^The selection procedure of hk markers is described in Supplementary Information 1.

### Development of population specific parental linkage maps and integrated Mukaku Kishu map

In ‘SB’ × ‘MK’ population, a total of 931 (817 ‘*lm* × *ll’* and 114 ‘*hk* × *hk’* markers) and 818 SNP markers (704 ‘*nn* × *np’* and 114 ‘*hk* × *hk’* markers) were used for constructing female (‘SB’) and male parent (‘MK_SB’) specific linkage maps (**Table 2**). From the total used markers, 930 for ‘SB’ and 810 for ‘MK_SB’ grouped on to nine major linkage groups (LGs) at independence LOD score of 6.0. The remaining markers formed small sub-groups and were not considered for grouping. Except two LGs in ‘SB’ map (LGs 1 and 8) and three in ‘MK_SB’ map (LGs 5, 6 and 8), all other LGs conserved their integrity up to LOD score of 10.0. The ‘SB’ map had 922 distinct locations and spanned a total map length of 1012.87 cM with an average marker interval of 1.09 cM. The ‘MK_SB’ map of this population had 807 unique locations and covered a genetic map length of 1165.72 cM (**Table 3**). The distribution of the skewed markers (*P =* 0.005) was not uniform across the LGs in two maps. In ‘SB’ map, LG2 had maximum of 96 distorted markers while LG9 contained 16 distorted markers. Similarly, in ‘MK_SB’ map, LG2 had maximum of 38 distorted markers and the number of distorted markers in other groups ranged from 1 to 31 (**Supplementary Table 2**).

**Table 3:** Summary of four parental maps and the integrated Mukaku Kishu (‘MK’) map.

Population	Parental Map	Total Mapped SNPs	Unique positions	Total syntenic SNPs	Number of distorted SNPs	Map Length (cM)	Average marker interval (cM)	Gaps (> 5 cM)
‘SB’ × ‘MK’	‘SB’	930 (9)^$^	922	916	129	1012.87	1.09	27
	‘MK_SB’	810 (9)	807	790	184	1165.72	1.44	27
‘D’ × ‘MK’	‘D’	776 (9)	734	754	125	879.37	1.13	23
	‘MK _D’	707 (9 + 1)	674	685	124	1018.57	1.44	18
Integrated	‘MK’	1233 (9)	1222	1201	299	1140.16	0.92	06

^$^The values in parenthesis denote to the linkage groups for the mapped SNPs on each map.

In ‘D’ × ‘MK’ population, 776 (664 *‘lm* × *ll’* and 112 ‘*hk* × *hk’* markers) and 710 SNP markers (598 ‘*lm* × *ll’* and 112 ‘*hk* × *hk’* markers) were used for constructing female (‘D’) and male (‘MK_D’) parent specific maps, respectively. In ‘D’ map, the 776 SNPs grouped into nine LGs at independent LOD threshold of 6.0. A further increase of LOD score caused splitting of the majority of LGs into sub-groups. The map had a total genetic length of 879.37 cM with an average marker interval of 1.13 cM (**Table 3**). For ‘MK_D’ map, 707 of 710 markers, grouped into nine major linkage groups and a minor linkage group. The minor LG contained 9 markers, of which 4 were the part of LG3 in ‘MK_SB’ map. Hence, the minor LG was presumed to be a part of the LG3 in this map. Like ‘D’ map, most of the LGs tended to lose their integrity in ‘MK_D’ map at LOD score > 6.0. The map consisted of 707 SNPs with 674 unique mapping points and covered a genetic length of 1018.57 cM. Like ‘SB’ × ‘MK’ population, the presence of skewed markers was also variable across LGs on these two maps. In ‘D’ map, LG8 had 30 distorted markers while LG5 and LG9 contained 22 and 26 such markers (**Supplementary Table 2**). In ‘MK_D’ map, except LG1 and LG2, all other LGs had distorted markers, and their number varied from 7 (LG6) to 44 (LG7) (**Supplementary Table 2**).

In both populations, the female maps contained more SNPs, but their overall map length was shorter than the corresponding male parent map. The linkage maps of ‘SB’ and ‘D’ had 120 and 69 more markers than the corresponding ‘MK’ maps but were 152.85 and 139.20 cM shorter in length, respectively. Except two LGs in ‘SB’ *×* ‘MK’ population (LGs 1 and 5) and two in ‘D’ *×* ‘MK’ population (LGs 4 and 5), all other LGs recorded higher ratio of male to female map length (**Supplementary Table 2**). Among the four parental maps, 18 (‘MK_D’) to 27 (‘SB’ and ‘MK_SB’) gaps of > 5 cM length were noted (**Table 3**).

The integrated ‘MK’ map was based on 1233 SNPs of which 284 (193 ‘*nn* × *np*’, 91 ‘*hk* ×*hk’*) were common between ‘MK_SB’ and ‘MK_D’ maps. The nine markers of subgroup 2 of LG 3 of ‘MK_D’ map easily integrated with homologous ‘MK_SB’ group to form integrated LG3. Due to increase in the number of markers, the average marker interval in the integrated ‘MK’ map was reduced to 0.92 from 1.44 in the two population specific ‘MK’ maps (**Table 3**). The integrated map had only six gaps of > 5 cM length (**Table 3**). The information of the detailed maps is provided in **Supplementary Table 2**.

### Development of sub-composite and consensus linkage maps

The use of heterozygous (‘*hk × hk’)* markers allowed building of sub-composite linkage maps between male and female parent specific maps in the two populations. In ‘SB’ × ‘MK’ population, the sub-composite linkage map (‘SB’-‘MK_SB’) was based on 1626 SNPs (816 ‘*lm × ll’*, 696 ‘*nn × np’* and 114 ‘*hk × hk’* markers) and spanned over a map length of 1105.14 cM with 1620 unique marker positions (**Table 4**). The average marker interval in the sub-composite ‘SB’-‘MK_SB’ map was 0.68 while for the individual LGs, it ranged from 0.59 (LG6) to 0.83 cM (LG1) (**Table 4**). Five gaps of > 5 cM were noted on this sub-composite map (**Table 4**) while one of them present on LG1 was even longer than 10 cM (data not shown).

**Table 4:** Attributes of two sub-composite and consensus mandarin maps.

Linkage group	Sub-composite maps										Consensus linkage map				
	‘SB’-‘MK_SB’ map					'D'-'MK-D' map									
	Total markers	Unique positions	Map length (cM)	Average marker interval (cM)	Gaps (> 5 cM)	Total markers	Unique positions	Map length (cM)	Average marker interval (cM)	Gaps (> 5 cM)	Total markers^#^	Unique positions	Map length (cM)	Average marker interval (cM)	Gaps (> 5 cM)
1	117	117	97.11	0.83	1	104	103	90.77	0.87	–	169	165	123.84	0.73	–
2	184	183	137.30	0.75	1	213	206	137.41	0.65	–	353	343	175.71	0.50	–
3	270	269	160.85	0.60	–	205	193	150.48	0.73	–	408	396	226.59	0.56	–
4	205	205	132.72	0.65	–	119	116	76.39	0.64	–	276	269	139.09	0.50	–
5	165	165	112.87	0.68	1	138	136	117.51	0.85	2	273	269	159.73	0.59	3
6	164	164	96.27	0.59	–	142	139	94.60	0.67	–	272	264	133.15	0.49	–
7	168	166	128.01	0.76	1	159	146	111.80	0.70	–	288	272	157.26	0.55	–
8	146	144	106.69	0.73	1	109	98	74.89	0.69	–	224	206	145.18	0.65	1
9	207	207	133.31	0.64	–	174	166	84.67	0.49	–	325	311	146.29	0.45	–
Total	1626	1620	1105.14	0.68	5	1363	1303	938.52	0.69	2	2588	2495	1406.84	0.54	4

^#^In consensus map, 401 of the total 2588 markers were common to both subs-composite maps. Details are given in **Supplementary Table 1**.

In ‘D’ × ‘MK’ population, the sub-composite map (‘D’-‘MK_D’) contained 1363 SNPs (664 ‘*lm × ll’*, 587 ‘*nn × np’* and 112 ‘*hk × hk’* markers). The nine markers of subgroup 2 of LG3 of ‘MK_D’ map (8 ‘*nn × np’* type and 1 ‘*hk × hk’* type) did not converge with homologous linkage group of female parent and were thus, not part of the sub-composite map. The mapped SNPs were present over 1303 distinct locations with a total genetic length of 938.52 cM. The average marker interval was 0.69 cM and for individual groups, it ranged from 0.49 (LG9) to 0.87 cM (LG1). Except two gaps of > 5 cM on LG5, the markers on all other LGs were evenly distributed (**Table 4**).

**Figure 3:**
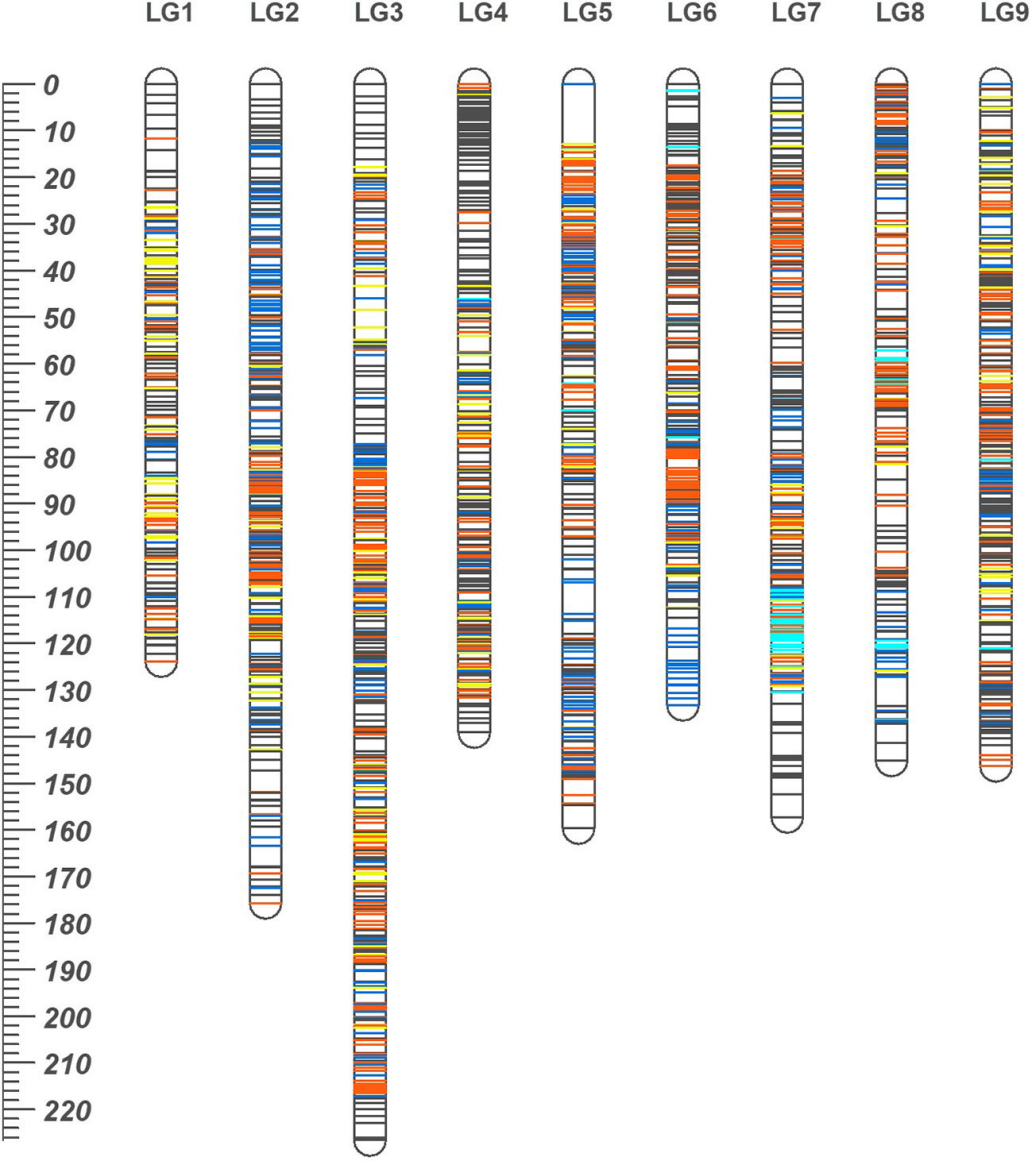
The high density consensus linkage map of mandarin. Nine linkage groups are numbered according to nine scaffolds of Clementine genome. On the map, *orange*, *blue* and *dark grey* lines represent marker sites specific to ‘SB’, Daisy (‘D’) and ‘MK’, respectively. The *yellow* lines represent the markers common to both ‘SB’ and ‘D’ while *cyan* lines are for markers translocated from other chromosomes with respect to their actual location on Clementine reference genome. The ruler at left indicates the map distances in cM.

The consensus linkage map was composed of 2588 markers (2587 SNPs and a phenotypic *Fs*-locus) of which 401 markers were shared between the two sub-composite maps. Of the shared markers, 211 between the maps of ‘SB’ and ‘D’ (120 ‘*lm × ll’* and 91 ‘*hk × hk’* markers), and 280 between the ‘MK_SB’ and ‘MK_D’ (190 ‘*nn × np’* and 90 ‘*hk × hk’* markers) were common (**Supplementary Table 1**). The common markers between ‘SB’ and ‘D’ have also been shown on the skeleton consensus map (**Figure 3**, **Supplementary Figure 1**). The consensus map had 2495 distinct positions and spanned a map length of 1406.84 cM. The marker density in the consensus linkage map was very high as the average marker to marker distance was 0.54 cM. The map length ranged from 123.84 cM for LG1 to 226.59 cM for LG3 (**Table 4**, **Figure 3**). The LG3 was also the largest linkage group in the two sub-composite maps.

In the consensus map, there were four gaps of > 5 cM (three on LG5 and one on LG8) (**Table 4**). One of the three gaps on LG5 was of more than 10 cM length (**Figure 2**, **Supplementary Figure 1**).

Based on the common SNP markers, the marker collinearity was also examined between the two sub-composite maps and consensus map. Except for very few markers, the order of most of the markers was consistent across the three maps (**Supplementary Figure 2**).

### Marker synteny

**Figure 4:**
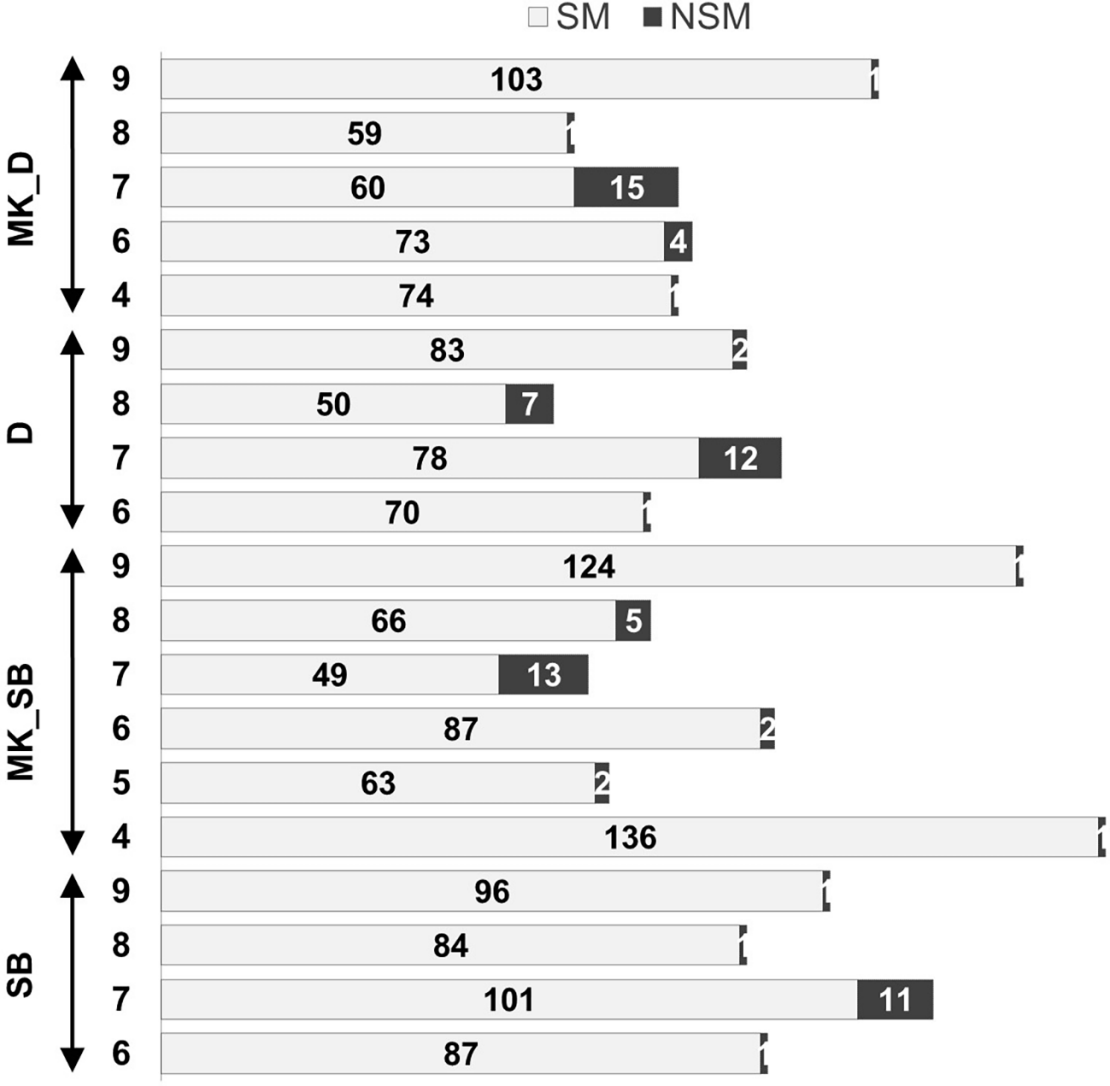
Non-syntenic linkage groups in the parental maps and proportion of syntenic (SM) and non-syntenic markers (NSM). ‘SB’, ‘MK_SB’ (Mukaku Kishu map derived from ‘SB’ × ‘MK’ population), ‘D’ and ‘MK_D’ (Mukaku Kishu map derived from ‘D’ × ‘MK’ population).

The grouping of the mapped SNPs showed high degree of synteny with their chromosomal location on the Clementine reference genome. Based on the number of syntenic markers, the parental maps of ‘SB’, ‘D’, ‘MK_SB’ and ‘MK_D’ shared 98.5, 97.2, 97.5 and 96.9% synteny with the Clementine reference genome. However, four LGs in both ‘D’ and ‘SB’ maps (LG6, LG7, LG8 and LG9), five LGs in ‘MK_D’ map (LG4, LG6, LG7, LG8 and LG9) and six LGs in ‘MK_SB’ map (LG4, LG5, LG6, LG7, LG8 and LG9) contained the markers translocated from other chromosomes relative to *C. clementina* reference genome (**Figure 4**). The LG7 showed maximum number of inter-chromosomal translocations in all of the parental maps and the consensus map (**Figures 3**, **4**).

### Mapping of seedlessness

**Figure 5:**
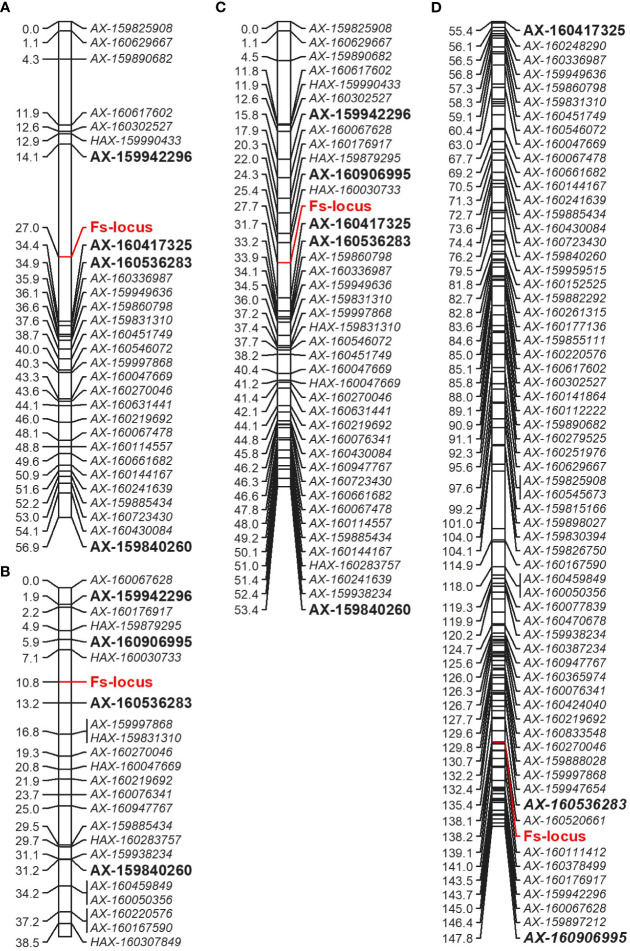
Comparative partial linkage map of LG5 of Mukaku Kishu derived from the ‘SB’ × ‘MK’ population (‘MK_SB’) **(A)**, ‘D’ × ‘MK’ population (‘MK_D’) **(B)**, integrated Mukaku Kishu from the two populations (‘MK’) **(C)**, and of consensus linkage map **(D)**. The markers showing linkage in the individual maps are bold and of large size. For easy comparison, the markers of female parents (‘SB’ and ‘D’) are not shown on consensus linkage map.

The *Fs*-locus was mapped on the LG5 in both individual ‘MK’ maps (‘MK_SB’ and ‘MK_D’) (**Figure 5**). It was linked to the SNP marker AX-160417325 at 7.4 cM on ‘MK_SB’ map. In ‘MK_D’ map, it localized between two SNP markers AX-160536283 and AX-160906995 at a distance of 2.4 and 4.9 cM, respectively. On the integrated ‘MK’ map of LG5, the three SNPs AX-160417325, AX-160536283, AX-160906995 maintained their proximity to the *Fs*-locus and were at a relative distance of 4.0, 5.5 and 3.4 cM distance (**Figure 5**). The proximity of *Fs*-locus with two of the three SNP markers was also supported by the high LOD value and relative low recombination frequency values during the grouping of different markers in the male parent maps (**Table 5**). The SNP marker AX-160536283 showed strong linkage with *Fs*-locus both in ‘MK_SB’ (LOD- 16.58, RF-0.09) and ‘MK_D’ (LOD- 14.02, RF-0.06) maps. On the other hand, the SNP marker AX-160417325 showed strong linkage with *Fs*-locus (LOD-17.4, RF-0.08) only in ‘MK_SB’ map (**Table 5**). Notably, AX-160417325 was used for mapping only in ‘SB’ × ‘MK’ population as it was an intercross marker for ‘D’ × ‘MK’ population with its allelic composition of T/T, T/C and T/C for ‘SB’, ‘D’ and ‘MK’, respectively.

**Table 5:** Proximity of SNP markers to *Fs*-locus in MK maps of different populations.

SNP markers	Attributes of linkage in different maps							
	‘MK_SB’		‘MK_D’		‘MK_L’		‘MK_CVO’	
	Recombination frequency	Linkage LOD	Recombination frequency	Linkage LOD	Recombination frequency	Linkage LOD	Recombination frequency	Linkage LOD
AX-160417325	0.0825	17.42	–	–	–	–		
AX-160536283	0.0928	16.58	0.0588	14.02	–	–		
AX-160906995	–	–	–	–	–	–		
AX-159840260	–	–	–	–	0.0000	3.96	0.0000	3.96
AX-159855111					0.0714	2.79	0.0714	2.79

On the consensus map, the closely linked SNP markers AX-160536283 and AX-160906995 were located at 2.8 cM and 9.6 cM from *Fs*-locus while the marker AX-160417325 mapped at a remote distance. The physical location of SNP markers AX-160417325 and AX-160536283 was explored on the Clementine annotated genome. The SNP markers AX-160417325 and AX-160536283 were located at 12.2 Mb and 10.0 Mb on the physical map of chromosome 5 (**Supplementary Information 2**). Thus, the physical map supported that the *Fs*-locus was more proximate to SNP marker AX160536283 than to AX-160417325.

The association of the *Fs*-locus with SNP markers was also independently examined for grouping statistics in two other F_1_ populations: ‘L’ × ‘MK’ (14 individuals) and ‘CVO’ × ‘MK’ (14 individuals). Here, none of the three closely linked markers showed proximity with the *Fs*-locus. The most closely associated SNP marker to the *Fs*-locus in these two populations was AX-159840260 (**Table 5**). This marker was at 29.9 and 20.4 cM in ‘MK_SB’ and ‘MK_D’ maps, respectively (**Figure 5**). Even the marker AX-160906995 located at 4.9 cM from *Fs*-locus on ‘MK_D’ map lacked direct proximity with it (**Table 5**).

### Evaluation of the selected SNPs to identify the seedless progenies

The two *Fs*-locus associated SNPs, AX-160417325 and AX-160536283, were assessed for predicting the seedless progenies in different populations (**Supplementary Table 3**). AX-160417325 was found to be the test cross marker for all the populations, except for ‘D’ × ‘MK’, where it behaved as an intercross marker. Its allelic pattern was T:T in maternal parents and T:C in the male parent ‘MK’. A selection based on the T:C allelic pattern, predicted 46 of the total 97 ‘SB’ × ‘MK’ cross progenies as seedless. Among the predicted 46, 40 were actual seedless progenies (true positives) and six were the false positives (seedy recombinants). Thus, the marker showed 87% PPV for seedless progeny detection in this population. The remaining two seedless progenies of this population exhibited the alternate allelic pattern and hence, were considered as false negatives. In the progenies of two minor populations (‘CVO’ × ‘MK’ and ‘T’ × ‘MK’), apart from the T:C allelic pattern, an improbable new C:C pattern was also found associated with seedlessness. Since the new variant carried allele ‘C’ from ‘MK’, we considered it linked with seedlessness. Based on T:C or C:C allelic patterns, all the seedless progenies could be identified in the three minor populations (**Supplementary Table 3**). However, abundant seedy individuals (false positives) also shared the seedless associated allelic patterns in these populations. Due to this reason, the AX-160417325 PPV for seedless progeny detection ranged from 25 to 50% in these populations. The AX-160536283 showed association with *Fs*-locus only in ‘SB’ × ‘MK’ and ‘D’ × ‘MK’ cross populations. It displayed 85.1 to 91.9% PPV for seedless progeny detection in these populations. The maternal (‘SB’ and ‘D’) and paternal (‘MK’) parents had the allelic patterns- A:A and A:G, respectively. Instead of ‘MK’ A:G allelic pattern, the majority of the seedless progenies had maternal A:A allelic pattern, indicating of its repulsive linkage with *Fs*-locus. The presence of some seedy individuals (false positives) among the sorted progenies in every population indicated that both markers are not co-segregating and that recombination is occurring in their vicinity (**Supplementary Table 3**). It is worth mentioning that there were gaps of 12.9 and 7.4 cM in the vicinity of the *Fs*-locus on ‘MK_SB’ map and of 2.4 and 4.9 cM on ‘MK_D’ map (**Figure 5**). Thus, to find the markers co-segregating with the *Fs*-locus, there is a need to bridge these gaps with additional polymorphic markers. However, the use of *Fs*-locus associated SNPs can reduce the effective population size preselected for seedlessness to be finally evaluated for all other fruit quality characteristics. The physical location of these SNPs on the Clementine genome and their flanking sequences are provided in **Supplementary Table 4**.

### Prediction of candidate genes for seedlessness

Based on the alignment of flanking SNP markers to the annotated Clementine genome, the *Fs*-locus on ‘MK_SB’ and ‘MK_D’ maps was delineated to a physical interval of 2.2 to 12.2 Mb and 3.97 to 10.0 Mb, respectively (**Figure 5** and **Supplementary Information 2**). These physical intervals house a total of 171 and 131 genes, respectively. The region of 3.97 to 10.0 Mb was common between the two maps which encompasses 131 genes. These genes are mainly related to floral and reproductive development, seed development, seed dormancy, seed germination, and are also involved in ABA signaling, and biotic and abiotic stress tolerance. In ‘MK’, the seedlessness is reportedly due to the developmental arrest of the embryos at an early stage. The functional exploration of the 131 genes across Arabidopsis and other crops helped in identification of 13 candidate genes which are reportedly expressed in developing embryos or seed coats (**Table 6**). These genes belong to seven gene families. One of these genes should be involved in imparting seedlessness in ‘MK’ and its derived seedless progenies.

**Table 6:** Prediction of putative candidate genes for seedlessness in ‘MK’.

Sr. No.	Gene ID	Position (bp)	Predicted function
1	Ciclev10003724m.g	4985850^#^-	UDP-Glycosyltransferase superfamily protein
2	Ciclev10003145m.g	4990017-	Wall associated kinase-like 1
3	Ciclev10000464m.g	5001178-5004379	Wall associated kinase-like 1
4	Ciclev10003390m.g	5037558-5047193	Wall associated kinase-like 1
5	Ciclev10003989m.g	5816248-5818999	Wall associated kinase-like 1
6	Ciclev10001614m.g	5921681-	Cytochrome P450, family 82, subfamily C, polypeptide 4
7	Ciclev10003405m.g	5938251-5940692	Cytochrome P450, family 82, subfamily C, polypeptide 3
8	Ciclev10003581m.g	6455788-6455828	ATP phosphoribosyl transferase 2
9	Ciclev10004097m.g	6458000-6461223	ATP phosphoribosyl transferase 2
10	Ciclev10003937m.g	6672504-	Homeobox protein 33
11	Ciclev10000860m.g	7064394-7068371	Cytochrome P450, family 706, subfamily A, polypeptide 6
12	Ciclev10000069m.g	8533088-8537312	T-complex protein 11
13	Ciclev10001947m.g	9480020-	6-phosphogluconate dehydrogenase family protein

^#^The genes mentioned without end positions had undefined length in the annotated reference Clementine genome.

## Discussion

High density linkage maps are essential for genetic analysis and mapping of economic traits. Apart from the factors of mapping population and their size (Ferreira et al., 2006; Li et al., 2010), use of high throughput whole genome genotyping methodologies is essential to develop high density linkage maps. High density citrus linkage maps have been generated for pummelo (Guo et al., 2015), sweet orange (Huang et al., 2018), *C. sunki* (Curtolo et al., 2018), and trifoliate orange (Curtolo et al., 2018; Huang et al., 2018) through high throughput whole genome genotyping methodologies using DArTseq markers (Curtolo et al., 2018) and SNP markers (Guo et al., 2015; Huang et al., 2018). The above methodologies were based on the simultaneous discovery and genotyping of markers over a population. These methodologies sometimes miss substantial genotypic information because of low sequencing depth (Gouesnard et al., 2017). The availability of whole genome sequences for a vast number of citrus accessions (Wu et al., 2014; Wu et al., 2018) has enabled discovery of abundant SNPs (Yu et al., 2016). The use of SNP probe sets as a fixed array may allow consistent genotyping for these SNPs across individuals and populations (Pandey et al., 2017), and minimize the possibility of missing information, and can lead to higher density genetic linkage maps. In this study, we evaluated the polymorphism content of the *Axiom_Citrus56 Array* (Hiraoka, 2020) over 210 individuals [206 F_1_ progenies derived from five different mandarin populations and four parent samples – LB8-9 Sugar Belle^®^ (‘SB’), Daisy (‘D’), Temple (‘T’) and Mukaku Kishu (‘MK’) (**Table 2**)]. The genotypic information of progenies of two populations: ‘SB’ × ‘MK’ and ‘D’ × ‘MK’, was used for constructing high density linkage maps for three mandarins- ‘SB’, ‘D’, and ‘MK’ and an integrated ‘MK’ map. The maps were built using SNP markers showing segregation pattern of test cross (‘*lm × ll’* and ‘*nn × np’* markers). The selected SNP markers segregating in 1:2:1 ratio (‘*hk* × *hk’* markers) (114 for ‘SB’ × ‘MK’ population and 112 for ‘D’ × ‘MK’ population) were also used in the dataset that allowed integration of male and female parent specific maps in the two maps. We also chose to use markers deviating from Mendelian pattern at significance level of *P* =0.005 with the assumption that these do not induce spurious linkage when coupled with independence LOD test statistic. The population specific sub-composite maps were further merged to construct a consensus mandarin map. The individual (‘MK_SB’, ‘MK_D’), integrated ‘MK’ map were used for deciphering the location of genes

### *Axiom_Citrus56 Array* and its polymorphism evaluation

Previously, a comparatively low density, 1536 SNP derived Illumina Golden Gate assay has been used in citrus for mapping fruit related traits of mandarins (Yu et al., 2016). The present array was the assembly of 58,433 SNP probe sets representing the SNP polymorphism of 41 citrus accessions, representing cultivated (different varieties of mandarin, sweet orange, grapefruit, pummelo, lime and lemons) and wild citrus (Hiraoka, 2020). The 40.2% of the SNPs (PHR and NMH) showed polymorphism for the set of 210 samples. Between the two mapping populations used in this study, percentage of polymorphic SNPs was substantially higher in ‘SB’ × ‘MK’ population (40.1%) relative to ‘D’ × ‘MK’ population (30.77%). Both ‘SB’ and ‘D’ are complex hybrids. The variability in polymorphism index may be due to different levels of heterozygosity in their genomes, resulting from variable degrees of admixture of the two progenitor species, *C. reticulata* Blanco and *C. maxima* [(Burm.) Merr] (Wu et al., 2014; Wu et al., 2018).

The polymorphism index of this array seemed to be lower than the previously reported high density SNP arrays in other fruit crops like pear (> 93% PHR SNPs) (Montanari et al., 2019) and apple (74% PHR and 2% NMH SNPs) (Bianco et al., 2016). The SNP frequencies tend to vary among crops, mainly influenced by their domestication history and reproductive habits. The original diversity of the samples under assessment, and the type (coding or non-coding) of the analyzed regions also influence the frequency of SNPs (Leonforte et al., 2013).

### Evaluation of map quality

The quality of the parental maps obtained in our study matched with other high quality linkage maps published for mandarins (Gulsen et al., 2010; Ollitrault et al., 2012). Like these maps, the markers were arranged on nine LGs in all the maps of our study (except for ‘MK_D’ map), corresponding to the haploid set of chromosomes of citrus. At LOD score of > 6.0, the affinity of the markers to remain grouped in different LGs was stronger in ‘SB’ × ‘MK’ population compared to ‘D’ × ‘MK’ population. This may be due to the more progeny individuals in ‘SB’ × ‘MK’ population, as grouping properties of markers are reported to improve significantly with increase of population size (Ferreira et al., 2006).

The average marker spacing on individual ‘SB’ (1.09 cM), ‘D’ (1.13 cM) and integrated ‘MK’ maps (0.92 cM) were comparable to the reference Clementine map reported by Ollitrault et al. (2012). The reference Clementine map was based on 961 co-dominant markers (677 SNPs, 258 SSRs and 26 indels) with total genetic length of 1084.1 cM and an average marker spacing of 1.13 cM. The markers number, map length and marker density was substantially higher over all other previously published maps for mandarins (Omura et al., 2000; Sankar and Moore, 2001; Oliveira et al., 2007; Gulsen et al., 2010; Curtolo et al., 2017).

Most of the mapped SNPs in the four parental maps showed a high degree of chromosomal synteny to the Clementine reference genome. The LG7 in all the four maps possessed the maximum number of translocations. This observation is consistent with the earlier findings of Huang et al. (2018) for molecular maps of trifoliate orange and sweet orange. The male ‘MK’ maps in both populations (‘MK_SB’ and ‘MK_D’) though, contained fewer segregating markers than the corresponding female maps, but had 15-16% greater map length. The variability of genetic distances among sex specific maps has also earlier been reported in citrus (Ollitrault et al., 2012; Huang et al., 2018). Ollitrault et al. (2012) reported lower recombination rates for the male compared to the female Clementine. Huang et al. (2018) observed greater map length for the pollen parent trifoliate orange compared to the seed parent sweet orange map. The mechanism of heterochiasmy *i.e.* the presence of differential cross over frequencies in male and female meiosis, may be responsible for this variability of genetic distances (Lenormand and Dutheil, 2005). According to this mechanism, gametic selection determines the heterochiasmy and the sex experiencing more stringent selection pressure during gametes production tend to have lower recombination frequencies. Bernet et al. (2010) in his experiments of cross pollination with compatible citrus parents found that the proportion of fertilized ovules is much greater than that of successful male gametes. In this study, the percentage of distorted markers was also greater in the male parent in both populations (**Supplementary Table 2**). Therefore, these results indicate that gametic selection is much more active in male than female gametes in citrus. The findings of the study are consistent with the recent reports of Garavello et al. (2020) in citrus. They studied the possible causes of segregation distortion by independently genotyping the pollen nuclei of the male parent [Clementine × sweet orange hybrid (‘CSO’)] and of the population progenies (‘RTSO’) resulting from the cross of female parent [(mandarin × sweet orange; ‘RTO’ tangor)] with ‘CSO’ pollen. The percentage segregation distortion was found to be lower in ‘CSO’ pollen than the ‘RTSO’ population nuclei. Hence, instead of the single sex based differential cross over frequency factor, the male gametes also experience selection pressure through other mechanisms like female-male gametic interactions or zygotic selection mechanisms (Garavello et al., 2020). From these other mechanisms, the S-RNase based gametophytic incompatibility system (GIS) causes male-female gamete interactions and contributes to segregation distortion in citrus (Garavello et al., 2020). GIS is the inability of a fertile hermaphrodite seed plant to produce zygotes after self-pollination. It has been found to be located on LG7 (Liang et al., 2019). In this study, ‘SB’ (Clementine mandarin × Minneola tangelo) and ‘D’ [(Clementine mandarin × Orlando tangelo) × (Clementine mandarin × Ponkan mandarin)] have Clementine as the common ancestor and therefore, could share an S-RNase haplotype, which could also be contributing towards this segregation distortion. Alternatively, the degree of heterozygosity of the parents has also been postulated to determine the rates of recombination and the genetic distance of maps. A higher degree of heterozygosity was found to correlate with lower recombination rates, as high heterozygosity suppresses recombination (Huang et al., 2018). The female parents used in the study are hybrids with relatively high degrees of heterozygosity, as a consequence of varying levels of admixture of two parental species, *C. reticulata* Blanco and *C. maxima* [(Burm.) Merr] (Wu et al., 2014; Wu et al., 2018). Mukaku Kishu is a somatic mutant of Kishu Mikan (*C. reticulata*) (Yamasaki et al., 2007), with less *C. maxima* introgression and therefore lower heterozygosity (Wu et al., 2018). Thus, multiple mechanisms could be the causes for the differences of genetic distance between male and female maps in this study.

### Distribution of distorted markers

The markers which deviate from Mendelian segregation ratios are referred to as distorted markers. The factors like statistical bias, errors during genotyping or scoring, or biological mechanisms have been proposed as possible causes of segregation distortion (Bradshaw and Stettler, 1994). The distribution of the distorted markers was not uniform across different linkage groups or the different maps. For instance, on LG2 of ‘SB’ map, LG7 of ‘MK_D’ map and LG8 of ‘D’ map, the proportion of the distorted markers was 87.3, 58.7 and 52.6%, respectively (**Supplementary Table 2**), but the distorted markers did not affect the grouping in our study as revealed by high LOD score for different linkage groups, thus suggesting that distortion may be due to a biological mechanism (Fishman et al., 2001). Similar observations were also noted in citrus by Ruiz and Asins (2003), and exclusion of such markers may result in a loss of significant information (Cervera et al., 2001). The selection operating in male or female gametes, their interactions with the cytoplasm, or differential selection of zygotic individuals are the possible biological mechanisms responsible for segregation distortion (Reflinur et al., 2014; Garavello et al., 2020; Ollitrault et al., 2021). The variable distribution of skewed markers on different LGs suggests that all these mechanisms were involved in distortion in the two populations of this study. For instance, the respective female maps ‘D’ and ‘SB’ had 12.8% and 87.3% skewed markers in LG2. On ‘MK_SB’ and ‘MK_D’ maps, this LG had 44.2% and no skewed markers, respectively (**Supplementary Table 2**). This highlights the possible role of female gametic selection, nuclear-cytoplasm interactions, and zygotic selection in segregation distortion in this LG (Reflinur et al., 2014).

### Consensus genetic map

Integration of maps across the parents (male and female) and populations is a useful approach to increase the marker density (Schlautman et al., 2017). The integration of maps is dependent upon the type of mapping population and the cross homology of the linkage groups. The use of intraspecific populations provides better opportunities to build integrated high density linkage maps compared to interspecific or intergeneric populations. Guo et al. (2015) successfully constructed an integrated map with 1543 SNP and 20 SSR markers using an intraspecific F_1_ population of 124 individuals. In contrast, Curtolo et al. (2017) attempted to construct a genetic map in an interspecific full-sib F_1_ population of 278 individuals derived from the cross of Murcott tangor and Pera sweet orange; only 661 SNP-based DArTseq markers were finally mapped on the integrated map.

In this study, we used two intraspecific mandarin populations generated using a common male parent Mukaku Kishu. This allowed us to prepare population specific, integrated male-female linkage maps (sub-composite maps) in JoinMap, followed by their merging into a consensus linkage map using MergeMap algorithm (Wu et al., 2008). MergeMap considers the marker order of individual maps to provide the consensus map order (Close et al., 2009). The consensus linkage map was based on 2588 markers (2587 distinct SNP markers and a phenotypic *Fs*-locus) positioned at 2495 locations and spanned over a genetic distance of 1406.84 cM. The reference Clementine map had an average marker density of 0.88 markers/cM (Ollitrault et al., 2012). The average marker density on the consensus map is 1.83 markers/cM with an average inter-marker distance of 0.54 cM. Thus, the present consensus map is a highly saturated map, and with an average genome size estimated to be 1500 to 1700 cM (Ollitrault et al., 1994), it covers 83 to 94% of the citrus genome. Due to high density of markers, high genome coverage and segregation information of two populations, the present map can serve as a future reference map for mandarins.

### Inheritance of seedlessness and prediction of candidate genes

Seedlessness is an important trait for fresh citrus scion breeding, and ‘MK’ is an attractive genetic resource for breeding seedless citrus. Previous reports showed that seedlessness in ‘MK’ and its seedless progenies was due to the arrest of embryo development at zygotic or pre-cotyledonary stage or due to formation of small seeds (Yamasaki et al., 2007; Yamasaki et al., 2009). Yamasaki et al. (2009) characterized the expression of seedlessness in ‘MK’ and its descendants. They found two types of seeds in ‘MK’ and its seedless descendants: ovule-like seeds without seed coat (1 mm in size), type A seeds (2-3 mm size) with an immature soft seed coat. They found that 10 weeks after pollination was a defining point in embryo development of different types of seeds. In either of the ‘MK’ type seeds, an arrest of embryo development or slow development was a common feature while the perfect seeds from seedy individuals had faster embryo development after this time point. Two genes are hypothesized to govern the seedlessness in ‘MK’; *Fs*, a dominant gene is proposed to support the seedlessness while *Is*, a repressor gene in dominant state inhibits seedless expression (Nesumi et al., 2001). The seedless ‘MK’ and seedy mandarins for these two genes were proposed to have the genetic constitution of *Fsfs-isis* and *fsfs-isis*, respectively (Nesumi et al., 2001; Yamasaki et al., 2007). Thus, in mandarin crosses with ‘MK’, a single gene could control the seedlessness. Based on this hypothesis, identification of RAPD markers linked to this *Fs*-locus was attempted earlier by Chavez and Chaparro (2011) using bulked segregant analysis (BSA) approach in the F_1_ progeny of GS (an open pollinated seedling selection of Robinson tangerine) and ‘MK’. They reported four RAPD markers, namely OPAI11-0.8, OPAJ19-1.0, OPM06r-0.85, and OPAJ04r-0.6, linked to the seedless locus at a distance of 4.3 to 8.7 cM. But, later these markers were found to be family specific. The results of RAPD markers are often found to be less repeatable (Li and Quiros, 2001) and are also sensitive to lab conditions. In this study, we mapped the seedless locus in two populations to identify and map the gene(s) governing the seedless traits. The putative candidate locus mapped on the LG5 of male parent ‘MK’. This observation is consistent with the findings of Shimada et al. (2014) who found seedlessness to be located on chromosome 5 of ‘Okitsu 46’ × ‘Kankitsu Chukanbohon Nou 5 gou’ map. The second parent ‘Kankitsu Chukanbohon Nou 5 gou’ in their study was a hybrid between ‘Lee’ (Clementine mandarin × Orlando tangelo) and ‘MK’. Their map was based on 708 EST-based CAPS markers, and the *Fs*-locus was mapped between markers Vs0015 and Edp005 at a distance of 7.2 and 3.1 cM, respectively. High-density maps can provide high resolution mapping of traits in one step and even preclude the need of fine mapping (Khan et al., 2012). In the present study, two markers AX-160536283 (at 2.4 cM on ‘MK_D’ map) and AX-160417325 (at 7.4 cM on ‘MK_SB’ map) showed close association with the *Fs*-locus. The first marker maintained its proximity in both populations, while the latter exhibited test cross segregation in the ‘SB’ × ‘MK’ population but appeared as an intercross marker for ‘D’ × ‘MK’ population. The consensus map also had AX-160536283 closer to the *Fs*-locus. The AX-160536283 showed 85.1 and 91.9% PPV for seedless progenies detection in ‘SB’ × ‘MK’ and ‘D’ × ‘MK’ populations, respectively. The AX-160417325 PPV for seedless progeny detection ranged from 25 to 87% in different observed populations. In two of the minor populations (‘CVO’ × ‘MK’ and ‘T’ × ‘MK’), an unexpected allelic constitution (C:C) was also observed in the seedless progenies in addition to the expected ‘MK’ T:C allelic pattern. This type of genotypic variation is a deviation from the expected Mendelian segregation, and is classified under the category of offspring Mendelian error. For most of the populations, the female parent and male parents were of T:T and T:C allelic patterns, the appearance of additional homozygotes for C allele (other than T:T) points to a case of an allele drop in (ADI_hom_) (Arias et al., 2022). This type of call in SNP array based genotyping may result from the presence of partial null allele in one of the parents. Since we did not observe such unexpected homozygotes in ‘SB’ × ‘MK’, ‘D’ × ‘MK’ and ‘L’ × ‘MK’ populations, such partial null allele in the remaining two minor populations is probably being passed from their female parents- ‘CVO’ and ‘T’ (Arias et al., 2022).

Based on the ‘MK_SB’ and ‘MK_D’ maps, the genomic region for the *Fs*-locus corresponded to a physical interval of 3.97-10.0 Mb between SNPs AX-160906995 and AX-160536283 on chromosome 5. This region encompasses 131 genes of which many are multiple copies (**Supplementary Information 2**). Seedlessness in ‘MK’ and its seedless progenies is due to the arrest of zygote growth from globular to pre-cotyledonary stage (pro-embryo stages) (Yamasaki et al., 2007). Thus, a gene which causes zygotic arrest at pro-embryo stage could be the potential candidate gene for imparting seedlessness in ‘MK’ and its progenies. From 131 genes, the 13 candidate genes representing seven gene families namely homeobox protein 33, wall associated kinase-like 1, cytochrome P450, T-complex proteins, 6-phosphoglucanate dehydrogenase, ATP phosphoribosyl transferase 2, and UDP-Glycosyltransferase superfamily protein are expressed in developing embryos or seed coat. Of these seven family genes, the homeobox gene is earlier reported to influence seed number in grapes (Li et al., 2019). Contrary to this, the ‘MK’ fruits are completely seedless (Yamasaki et al., 2007). The genes encoding cytochrome P450 genes and wall associated proteins have a specific expression pattern in embryo sac units and embryos (Wang et al., 2012; Sotelo-Silveira et al., 2013). The ATP phosphoribosyl transferase 2 is involved in histidine biosynthesis, which is essential for normal embryo development; its mutation reportedly caused embryo abortion in Arabidopsis (DeFraia and Leustek, 2004). DeFraia and Leustek (2004) found that the wild types (homozygous dominant for this gene) could produce sufficient histidine for embryo growth while the heterozygous mutants had insufficient histidine to support embryo development, eventually leading to their abortion. The UDP-Glycosyltransferase superfamily protein is expressed in seed coats post-torpedo stage (Barvkar et al., 2012). Any mutation in the T-complex protein coding gene is known to cause impaired embryo development (Garcia et al., 2017). The 6-phosphoglucanate dehydrogenase genes code for an enzyme which is involved in oxidative pentose phosphate pathway. The plastidial pentose phosphate pathway is essential for post-globular stage development of embryos in Arabidopsis (Andriotis and Smith, 2019). Any defect or mutation in the above-described genes should be imparting complete seedlessness in ‘MK’ and its seedless progenies.

In this study, we found the location of the *Fs*-locus on LG5 of ‘MK’ (Mukaku Kishu) and identified two closely associated SNPs, AX-160417325 and AX-160536283. These SNPs reduced the effective population size and positively predicted seedlessness in 25.0-91.9% of the progenies in studied populations. These markers should prove useful for reducing the effective population size at seedling stage in crosses involving ‘MK’ paternity. Yet, the presence of seedless allelic pattern in some seedy individuals (false positives) and very few seedless individuals sharing the alternate allelic pattern indicate that these are not co-segregating markers. There are some gaps in immediate vicinity of *Fs*-locus in both ‘MK_SB’ and ‘MK_D’ maps. To underpin the exact causative gene and find a co-segregating marker for this trait, the region need to be delimited with use of additional cross populations, increase in size of the mapping population, and inclusion of additional polymorphic markers. Further, complementation of the study with expression analysis could assist in confirming the gene governing the seedlessness in Mukaku Kishu.

## Data availability statement

The original contributions presented in the study are included in the article/**Supplementary Material**, further inquiries can be directed to the corresponding author/s.

## Author contributions

FG and QY conceived and designed the study. KK and CH performed the experiment. KK, DB, and QY performed data analysis and prepared genetic maps. KK, DB, and QY wrote the rough draft of manuscript. FG and QY supervised the project. FG managed funds and revised the manuscript. All authors contributed to the article and approved the submitted version.
