# Supplementary material for: Construction of a high density genetic linkage map to define the locus conferring seedlessness from Mukaku Kishu mandarin
Source: Front Plant Sci. 2023 Feb 14;14:1087023. doi: 10.3389/fpls.2023.1087023 (PMC9976630; doi:10.3389/fpls.2023.1087023)
Supplement: Supplementary file 1 [file Presentation_1.pptx]

## Slide 1
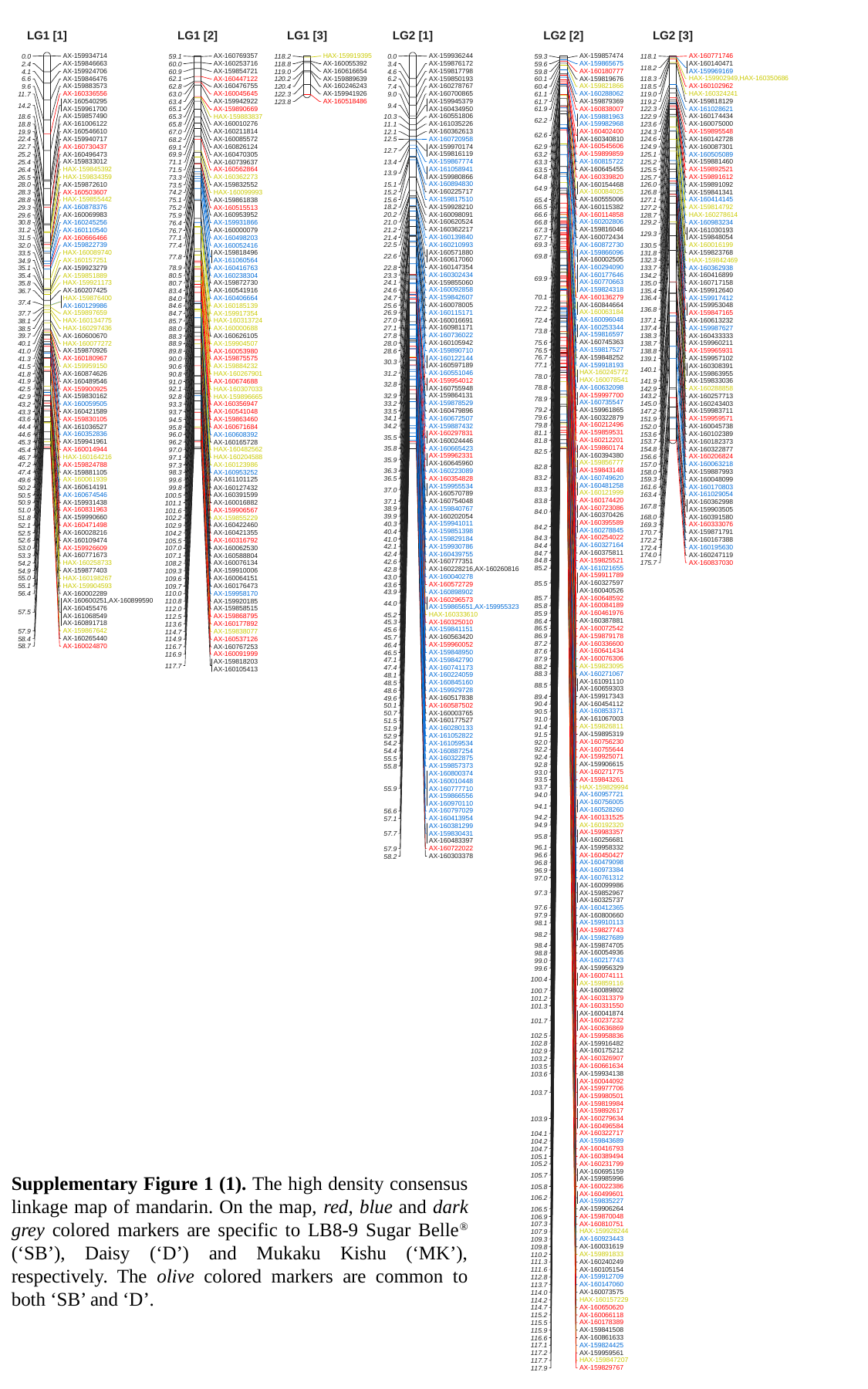

Supplementary Figure 1 (1). The high density consensus linkage map of mandarin. On the map, red, blue and dark grey colored markers are specific to LB8-9 Sugar Belle® (‘SB’), Daisy (‘D’) and Mukaku Kishu (‘MK’), respectively. The olive colored markers are common to both ‘SB’ and ‘D’.

## Slide 2
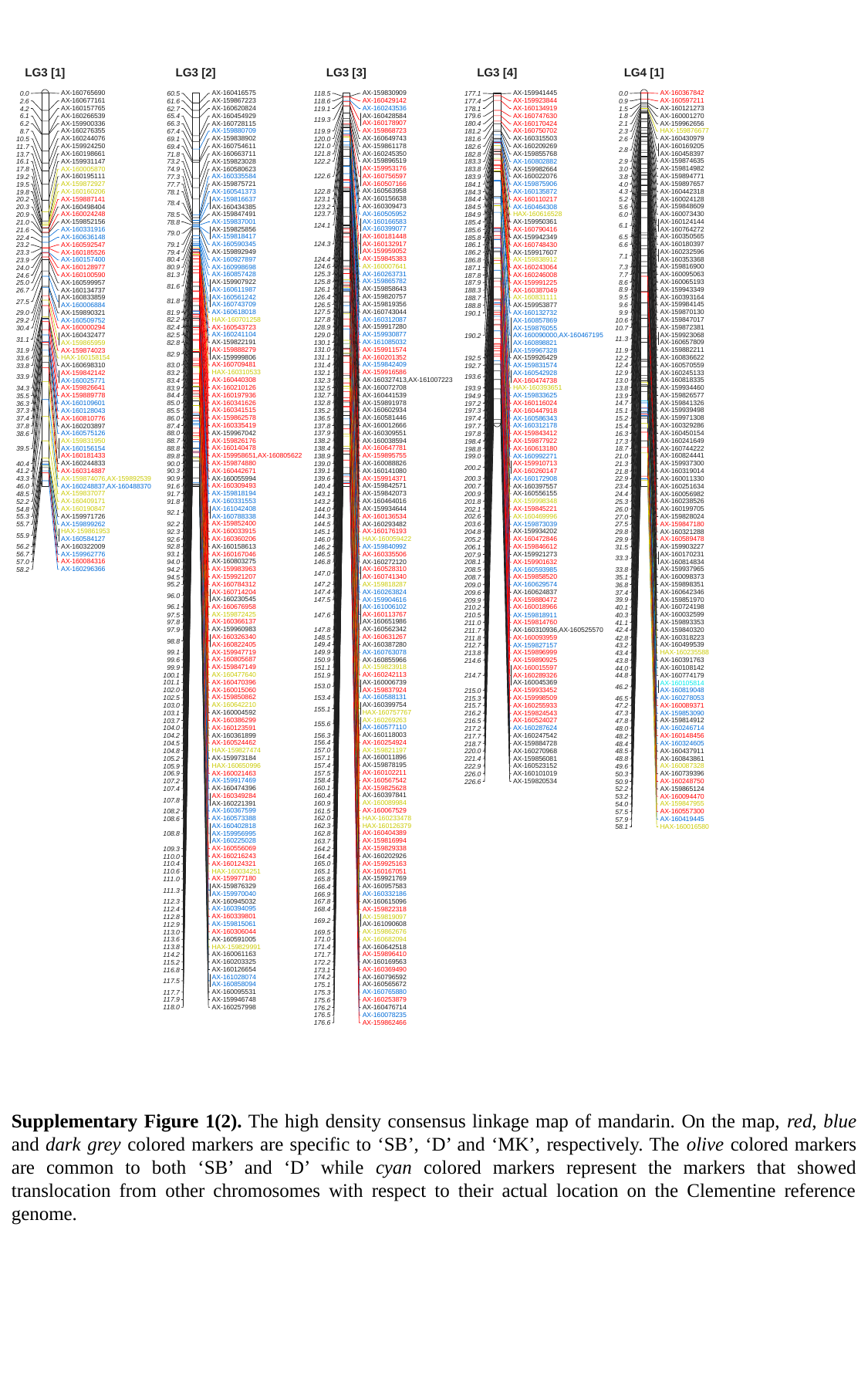

Supplementary Figure 1(2). The high density consensus linkage map of mandarin. On the map, red, blue and dark grey colored markers are specific to ‘SB’, ‘D’ and ‘MK’, respectively. The olive colored markers are common to both ‘SB’ and ‘D’ while cyan colored markers represent the markers that showed translocation from other chromosomes with respect to their actual location on the Clementine reference genome.

## Slide 3
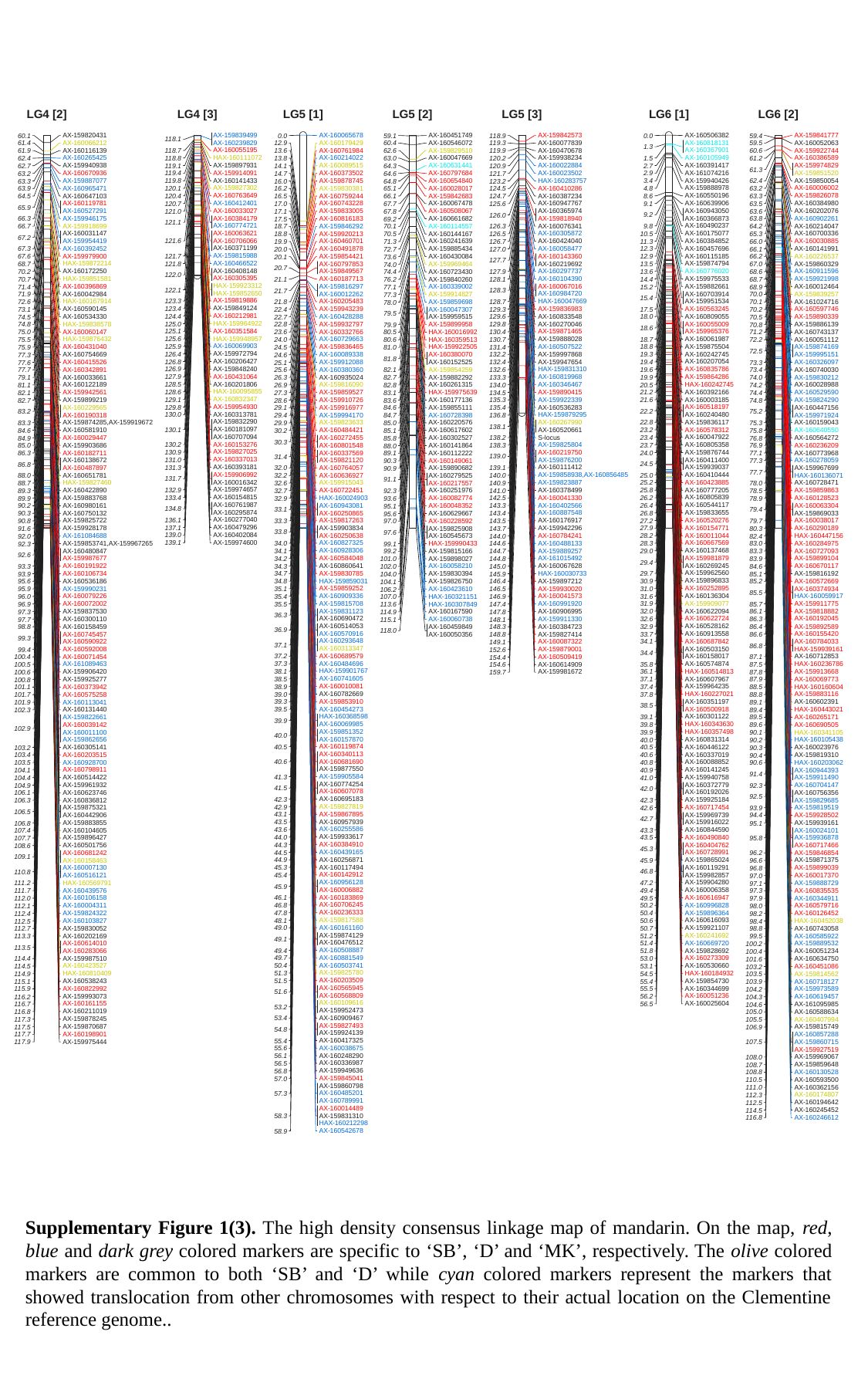

Supplementary Figure 1(3). The high density consensus linkage map of mandarin. On the map, red, blue and dark grey colored markers are specific to ‘SB’, ‘D’ and ‘MK’, respectively. The olive colored markers are common to both ‘SB’ and ‘D’ while cyan colored markers represent the markers that showed translocation from other chromosomes with respect to their actual location on the Clementine reference genome..

## Slide 4
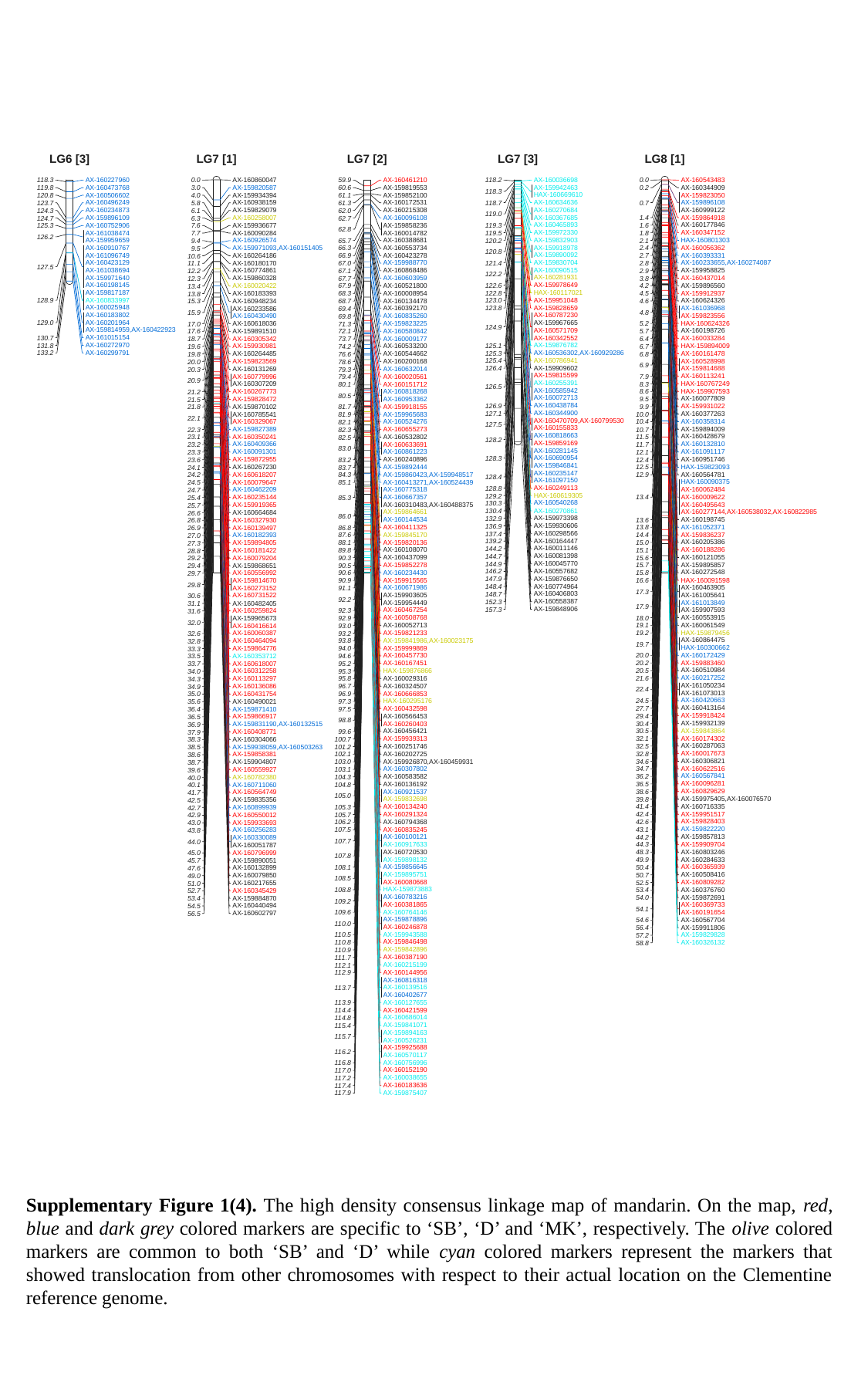

Supplementary Figure 1(4). The high density consensus linkage map of mandarin. On the map, red, blue and dark grey colored markers are specific to ‘SB’, ‘D’ and ‘MK’, respectively. The olive colored markers are common to both ‘SB’ and ‘D’ while cyan colored markers represent the markers that showed translocation from other chromosomes with respect to their actual location on the Clementine reference genome.

## Slide 5
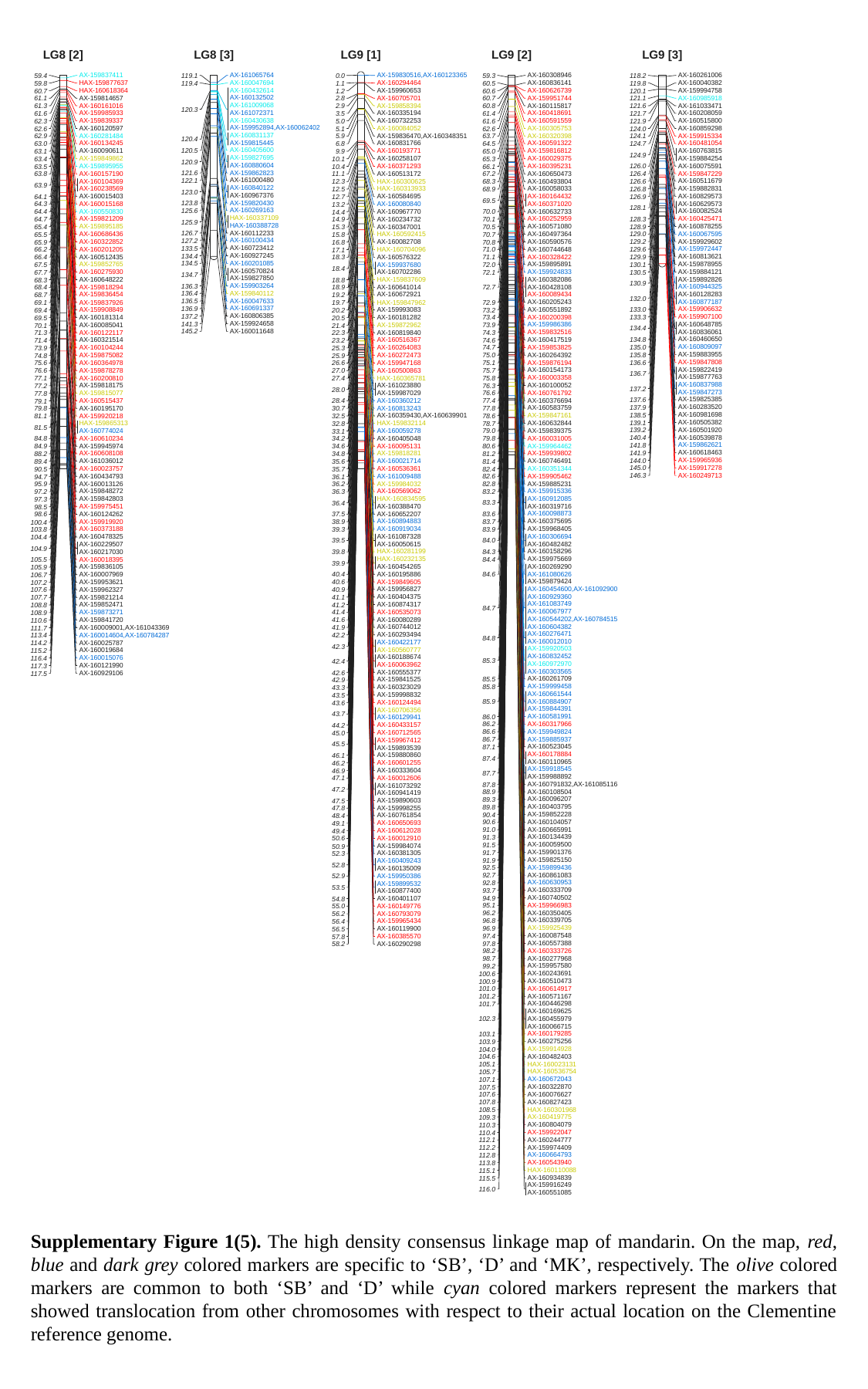

Supplementary Figure 1(5). The high density consensus linkage map of mandarin. On the map, red, blue and dark grey colored markers are specific to ‘SB’, ‘D’ and ‘MK’, respectively. The olive colored markers are common to both ‘SB’ and ‘D’ while cyan colored markers represent the markers that showed translocation from other chromosomes with respect to their actual location on the Clementine reference genome.
