# Supplementary material for: Construction of a high density genetic linkage map to define the locus conferring seedlessness from Mukaku Kishu mandarin
Source: Front Plant Sci. 2023 Feb 14;14:1087023. doi: 10.3389/fpls.2023.1087023 (PMC9976630; doi:10.3389/fpls.2023.1087023)
Supplement: Supplementary file 2 [file Presentation_2.pptx]

## Slide 1
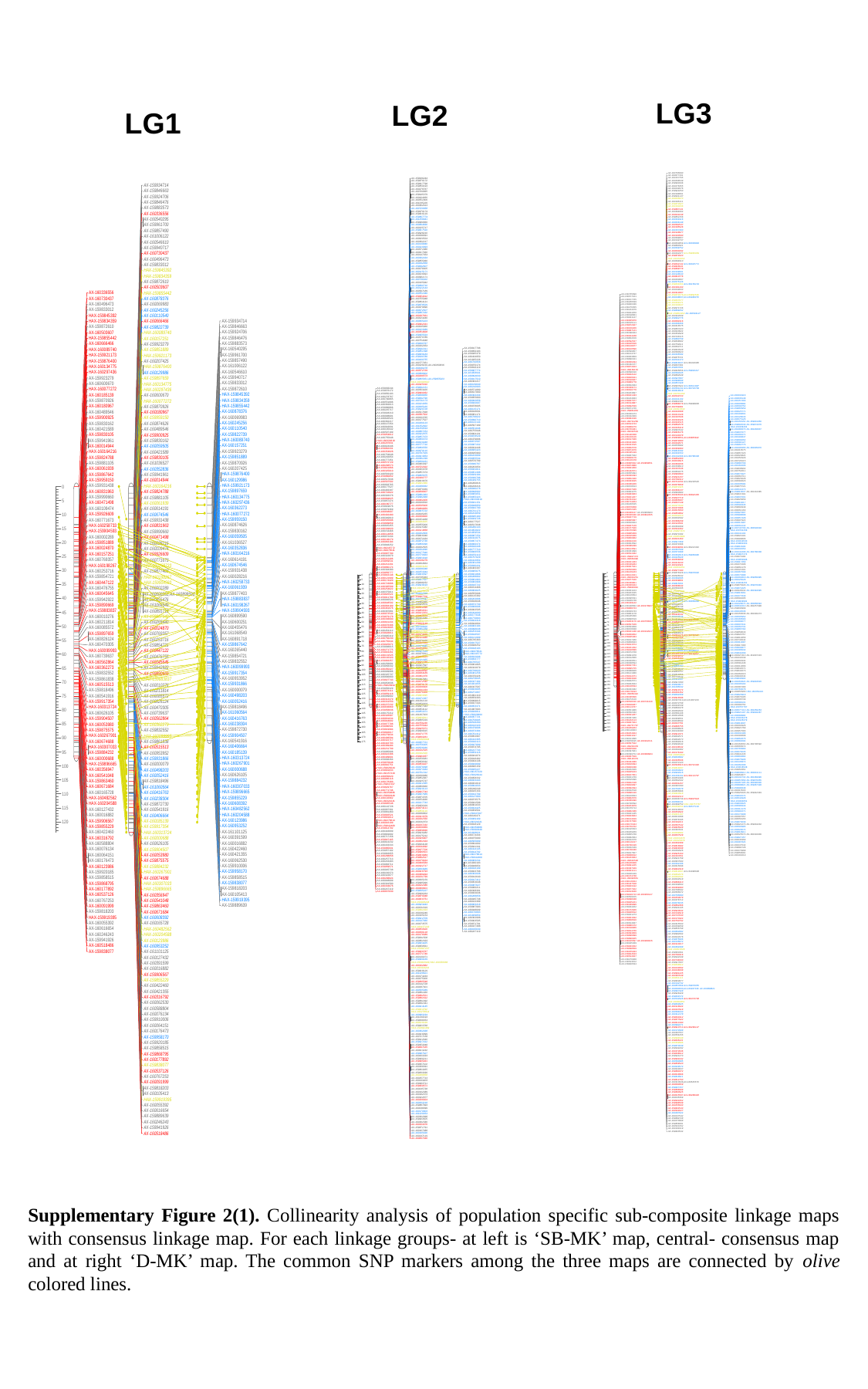

LG3
LG2
LG1
Supplementary Figure 2(1). Collinearity analysis of population specific sub-composite linkage maps with consensus linkage map. For each linkage groups- at left is ‘SB-MK’ map, central- consensus map and at right ‘D-MK’ map. The common SNP markers among the three maps are connected by olive colored lines.

## Slide 2
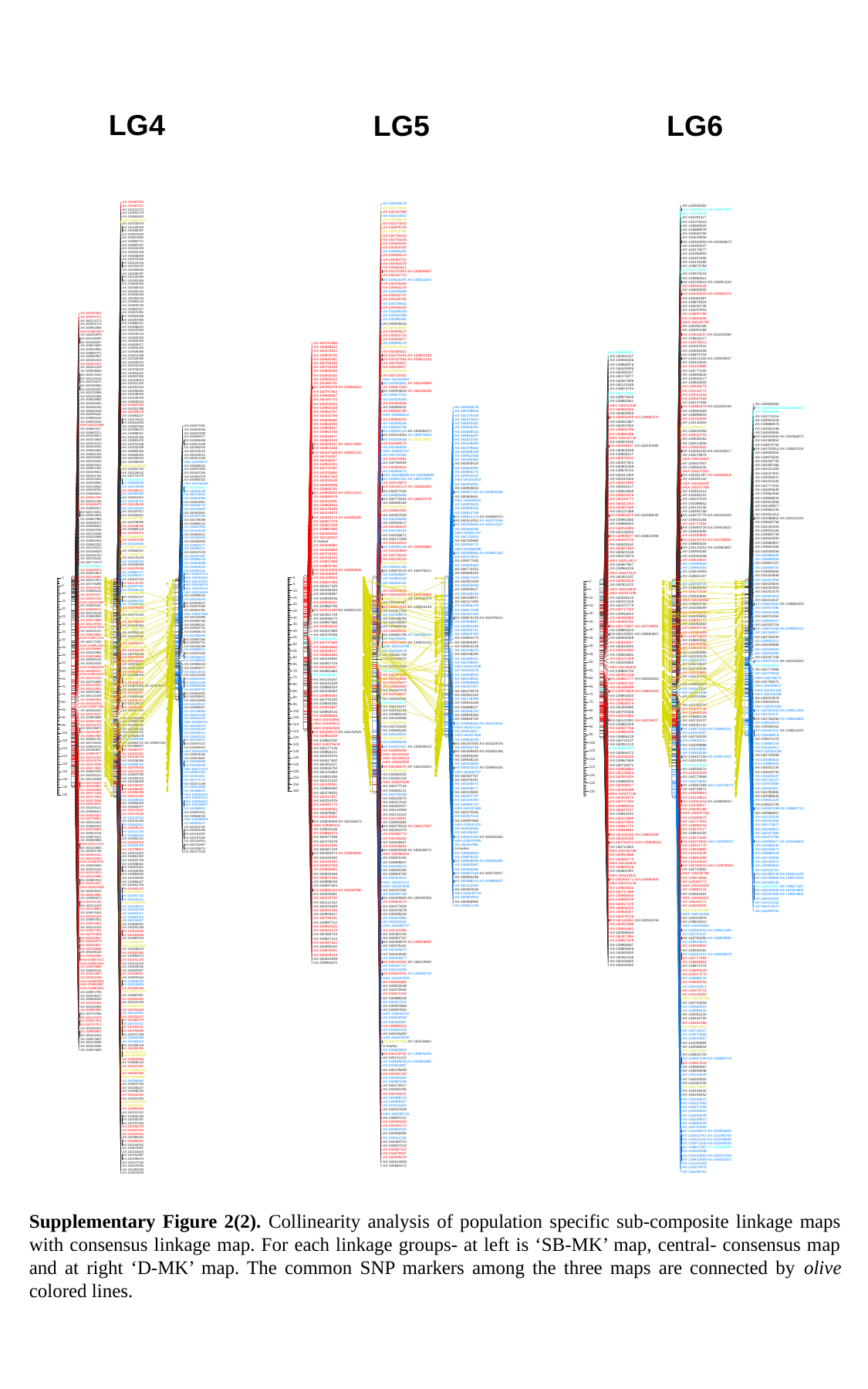

LG4
LG5
LG6
Supplementary Figure 2(2). Collinearity analysis of population specific sub-composite linkage maps with consensus linkage map. For each linkage groups- at left is ‘SB-MK’ map, central- consensus map and at right ‘D-MK’ map. The common SNP markers among the three maps are connected by olive colored lines.

## Slide 3
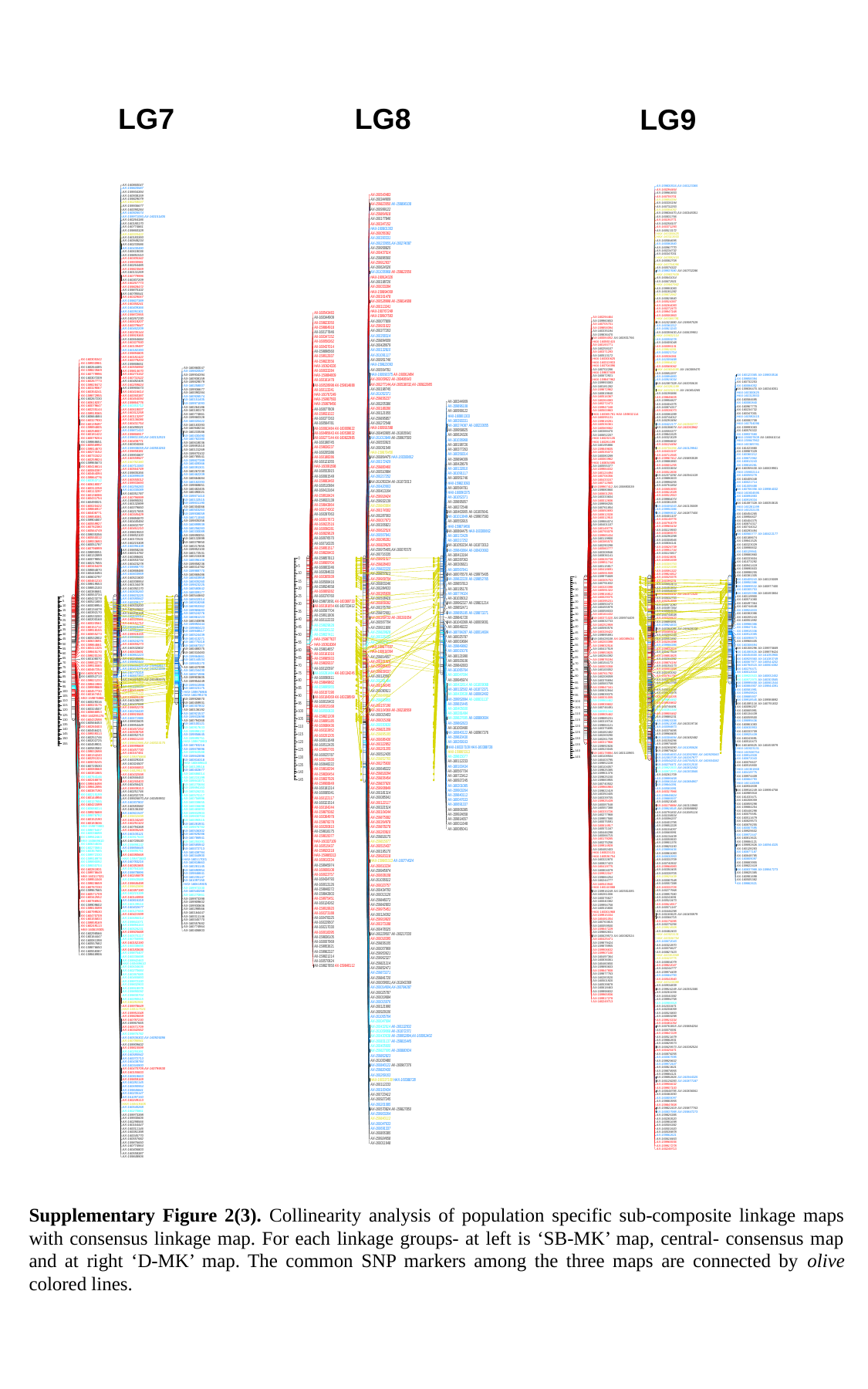

LG7
LG8
LG9
Supplementary Figure 2(3). Collinearity analysis of population specific sub-composite linkage maps with consensus linkage map. For each linkage groups- at left is ‘SB-MK’ map, central- consensus map and at right ‘D-MK’ map. The common SNP markers among the three maps are connected by olive colored lines.
