# Supplementary material for: Construction of a high density genetic linkage map to define the locus conferring seedlessness from Mukaku Kishu mandarin
Source: Front Plant Sci. 2023 Feb 14;14:1087023. doi: 10.3389/fpls.2023.1087023 (PMC9976630; doi:10.3389/fpls.2023.1087023)
Supplement: Supplementary file 3 [file Table_1.docx]

**Supplementary Table 1.** Details of the total and common SNPs mapped in ‘SB’ × ‘MK’ and ‘D’ × ‘MK’ populations.

| **Linkage group** | **‘SB’ × ‘MK’ population** | | | **‘D’ × ‘MK’ population** | | | **Markers common between two populations** | | | |
| --- | --- | --- | --- | --- | --- | --- | --- | --- | --- | --- |
|  | ***lm ×ll* type** | ***nn × np* type** | **Common *hk ×hk* type** | ***lm ×ll* type** | ***nn× np* type** | **Common *hk ×hk* type** | **Common *lm ×ll* markers** | **common *nn×np* markers** | **Common *hk ×hk* markers** | **Total** |
| 1 | 47 | 48 | 22 | 33 | 49 | 22 | 15 | 15 | 22 | 52 |
| 2 | 98 | 74 | 12 | 120 | 81 | 12 | 13 | 19 | 12 | 44 |
| 3 | 165 | 91 | 14 | 116 | 75+8^$^ | 13+1^$^=14 | 26 | 27+3^$^ | 13+1^$^=14 | 67+3^$^ |
| 4 | 68 | 120 | 17 | 44 | 58 | 17 | 09 | 22 | 17 | 48 |
| 5 | 100 | 60 | 05 | 83 | 43 | 12 | 16 | 14 | 00 | 30 |
| 6 | 75 | 76 | 13 | 65 | 71 | 06 | 08 | 24 | 02 | 34 |
| 7 | 106 | 56 | 06 | 84 | 69 | 06 | 14 | 19 | 06 | 39 |
| 8 | 75 | 61 | 10 | 49 | 52 | 08 | 06 | 22 | 03 | 31 |
| 9 | 82 | 110 | 15 | 70 | 89 | 15 | 13 | 28 | 15 | 56 |
| **Total** | **816** | **696** | **114** | **664** | **595** | **112** | **120** | **190+3** | **91** | **401+3^$^** |

^$^Denotes to three markers from subgroup 2 of ‘D’ LG3, which were not present in sub-composite and consensus map.
