# Supplementary material for: Construction of a high density genetic linkage map to define the locus conferring seedlessness from Mukaku Kishu mandarin
Source: Front Plant Sci. 2023 Feb 14;14:1087023. doi: 10.3389/fpls.2023.1087023 (PMC9976630; doi:10.3389/fpls.2023.1087023)
Supplement: Supplementary file 4 [file Table_2.docx]

**Supplementary Table 2.** Details of component linkage maps of parents-**‘SB’, ‘MK_SB’**, **‘D’, ‘MK_D’** the two populations and integrated ‘MK’ map.

| **Linkage Group** | **‘SB’ × ‘MK’ population** | | | | | | | | | | **‘D’ × ‘MK’ population** | | | | | | | | | | **Integrated ‘MK’ (MK_SB +MK_D)** | | | | |
| --- | --- | --- | --- | --- | --- | --- | --- | --- | --- | --- | --- | --- | --- | --- | --- | --- | --- | --- | --- | --- | --- | --- | --- | --- | --- |
|  | **‘SB’** | | | | | **‘MK_SB’** | | | | | **‘D’** | | | | | **‘MK_D’** | | | | |  |  |  |  |  |
|  | **Total mar-kers** | **No. of distorted markers** | **Map length (cM)** | **Inter locus interval** | **Gaps**  **(> 5**  **cM)** | **Total mar-kers** | **No. of distorted markers** | **Map length (cM)** | **Inter locus interval** | **Gaps**  **(> 5 cM)** | **Total mar-kers** | **No. of distorted markers** | **Map length (cM)** | **Inter locus interval** | **Gaps**  **(> 5 cM)** | **Total mar-kers** | **No. of distorted markers** | **Map length**  **(cM)** | **Inter locus interval** | **Gaps**  **(> 5 cM** | **Total mar-kers** | **No. of distorted markers** | **Map length**  **(cM)** | **Inter locus interval** | **Gaps**  **(> 5 cM** |
| 1 | 69 | 02 | 95.64 | 1.39 | 1 | 70 | 01 | 88.19 | 1.26 | 0 | 55 | 01 | 90.08 | 1.64 | 2 | 71 | 00 | 97.25 | 1.37 | 1 | 104 | 01 | 100.63 | 0.97 | 0 |
| 2 | 110 | 96 | 104.26 | 0.95 | 4 | 86 | 38 | 141.64 | 1.65 | 3 | 132 | 17 | 128.05 | 0.97 | 3 | 93 | 00 | 151.53 | 1.63 | 4 | 148 | 38 | 146.28 | 0.99 | 0 |
| 3 | 179 | 00 | 129.08 | 0.72 | 0 | 105 | 19 | 199.92 | 1.90 | 8 | 130 | 14 | 139.89 | 1.08 | 3 | 88+9^$^ | 10+9 | 157.26 | 1.62 | 2 | 158 | 34 | 191.82 | 1.21 | 1 |
| 4 | 85 | 01 | 113.80 | 1.34 | 2 | 137 | 09 | 137.35 | 1.00 | 2 | 61 | 05 | 90.61 | 1.49 | 5 | 75 | 17 | 85.80 | 1.14 | 0 | 173 | 26 | 113.30 | 0.65 | 1 |
| 5 | 105 | 05 | 131.37 | 1.25 | 5 | 64+*Fs*-locus= 65 | 06 | 115.17 | 1.77 | 5 | 95 | 22 | 120.35 | 1.27 | 3 | 54+ *Fs*-locus  = 55 | 16 | 90.18 | 1.64 | 2 | 106 | 21 | 112.81 | 1.06 | 1 |
| 6 | 88 | 04 | 90.82 | 1.03 | 4 | 89 | 35 | 93.49 | 1.05 | 0 | 71 | 01 | 67.21 | 0.95 | 1 | 77 | 07 | 83.06 | 1.08 | 0 | 140 | 40 | 91.33 | 0.65 | 0 |
| 7 | 112 | 00 | 105.41 | 0.94 | 3 | 62 | 21 | 132.12 | 2.13 | 4 | 90 | 09 | 88.71 | 0.99 | 3 | 75 | 44 | 115.88 | 1.55 | 3 | 112 | 65 | 138.01 | 1.23 | 2 |
| 8 | 85 | 05 | 109.25 | 1.29 | 5 | 71 | 24 | 124.55 | 1.75 | 5 | 57 | 30 | 66.34 | 1.16 | 2 | 60 | 08 | 108.79 | 1.81 | 5 | 106 | 32 | 119.85 | 1.13 | 1 |
| 9 | 97 | 16 | 133.24 | 1.37 | 3 | 125 | 31 | 133.30 | 1.07 | 0 | 85 | 26 | 88.13 | 1.04 | 1 | 104 | 13 | 128.83 | 1.24 | 1 | 186 | 42 | 126.14 | 0.68 | 0 |
| **Total** | **930** | **129** | **1012.87** | **1.09** | **27** | **810** | **184** | **1165.72** | **1.44** | **27** | **776** | **125** | **879.37** | **1.13** | **23** | **707** | **124** | **1018.57** | **1.44** | **18** | **1233** | **299** | **1140.16** | **0.92** | **6** |

^$^The LG 2 was arranged in two sub-groups in ‘MK_D’
