# Supplementary material for: Construction of a high density genetic linkage map to define the locus conferring seedlessness from Mukaku Kishu mandarin
Source: Front Plant Sci. 2023 Feb 14;14:1087023. doi: 10.3389/fpls.2023.1087023 (PMC9976630; doi:10.3389/fpls.2023.1087023)
Supplement: Supplementary file 5 [file Table_3.docx]

**Supplementary Table 3.** Evaluation of the closely linked SNPs for marker assisted selection (MAS) of seedless progenies in different populations.

| Popul-ation | SNP allelic pattern in parents | Seed phenotype | | SNP genotyping of the progeny | | | Evaluation of AX-160417325 and  AX-160536283 for MAS | | | | |
| --- | --- | --- | --- | --- | --- | --- | --- | --- | --- | --- | --- |
|  |  | Class | Number | Pattern 1 | Pattern 2 | Other pattern | Total progenies sorted | SL | False positives | False negatives | Positive predictive value (PPV) (%) |
| AX-160417325 | | | | T:C | T:T | C:C |  | | | |  |
| ‘SB’  × ‘MK’ | T:T × T:C | SL^##^ | 42 | 40 | 2 | - | 46 | 40 | 6 | 2 | 87.0 |
|  |  | S | 55 | 6 | 49 | - |  |  |  |  |  |
|  |  | Total | 97 | 46 | 51 | 0 |  |  |  |  |  |
| ‘CVO’^#^ × ‘MK’ | -- × T:C | SL | 5 | 3 | - | 2 | 12 | 5 | 7 | 0 | 41.7 |
|  |  | S | 9 | 7 | 2 | - |  |  |  |  |  |
|  |  | Total | 14 | 10 | 2 | 2 |  |  |  |  |  |
| ‘L’^#^  × ‘MK’ | -- × T:C | SL | 1 | 1 | - | - | 4 | 1 | 3 | 0 | 25.0 |
|  |  | S | 13 | 3 | 10 | - |  |  |  |  |  |
|  |  | Total | 14 | 4 | 10 | 0 |  |  |  |  |  |
| ‘T’  × ‘MK’ | T:T × T:C | SL | 4 | 3 | - | 1 | 8 | 4 | 4 | 0 | 50.0 |
|  |  | S | 9 | 4 | 5 | - |  |  |  |  |  |
|  |  | Total | 13 | 7 | 5 | 1 |  |  |  |  |  |
| AX-160536283 | | | | A:G | A:A | -- |  |  |  |  |  |
| ‘SB’  × ‘MK’ | A:A × A:G | SL | 42 | 2 | 40 | - | 47 | 40 | 7 | 2 | 85.1 |
|  |  | S | 55 | 48 | 7 | - |  |  |  |  |  |
|  |  | Total | 97 | 50 | 47 | - |  |  |  |  |  |
| ‘D’  × ‘MK’ | A:A × A:G | SL | 35 | 1 | 34 |  | 37 | 34 | 3 | 1 | 91.9 |
|  |  | S | 33 | 30 | 3 |  |  |  |  |  |  |
|  |  | Total | 68 | 31 | 37 |  |  |  |  |  |  |

^#^The female parents ‘CVO’ and Lee (‘L’) mandarins were not the part of our SNP array-based analysis. ^##^SL and S indicates the seedless and seedy progenies in the respective population.
