# Supplementary material for: Construction of a high density genetic linkage map to define the locus conferring seedlessness from Mukaku Kishu mandarin
Source: Front Plant Sci. 2023 Feb 14;14:1087023. doi: 10.3389/fpls.2023.1087023 (PMC9976630; doi:10.3389/fpls.2023.1087023)
Supplement: Supplementary file 7 [file Table_4.docx]

**Supplementary Table 4.** Physical location of the SNP markers, and their flanking sequences for marker assisted selection.

| SNP marker | Location on the physical map (Mb) | DNA sequence flanking the SNP site (5’-3’) |
| --- | --- | --- |
| AX-160417325 | 12207492 | ACTTTATGTGGTGGTCGAATCCTTCTCCTTTAAGA[C/T]GTTTTCTACACAATTAATCTTTTGGGAAAATTACC |
| AX-160536283 | 10002522 | CAATTGTACTGGCTCCTTTCTTTCCATTTCAAAAC[A/G]TAGGGTGTATGGTACTGATTGATAGCTTTATGTAT |
