# Supplementary material for: Construction of a high density genetic linkage map to define the locus conferring seedlessness from Mukaku Kishu mandarin
Source: Front Plant Sci. 2023 Feb 14;14:1087023. doi: 10.3389/fpls.2023.1087023 (PMC9976630; doi:10.3389/fpls.2023.1087023)
Supplement: Supplementary file 8 [file Table_5.docx]

**Supplementary information 1: Processing of heterozygous SNPs (‘*hk’* × ‘*hk’ markers*)**

**STEPS USED:**

1. After filtering the markers dataset through Axiom analysis, 2539 heterozygous SNP (*‘hk’*) markers in Daisy (‘D’) × Mukaku Kishu (‘MK’) population and 1854 hk markers in Sugar Belle (‘SB’) × ‘MK’ population were obtained.
2. After comparison, the common 1147 hk markers were selected.
3. These 1147 *hk* markers were processed with ‘MK’ (*‘nn’* × *‘np’*) dataset of Daisy × Mukaku Kishu population in JoinMap software.
4. 121 *hk* markers were obtained after eliminating the markers with 95% or higher similarity and those deviating from 1:2:1 segregation at significance level of 0.5%. The 121 hk markers were processed with male (*‘nn’* × *‘np’*) and female (*‘lm’* × *‘ll’*) specific marker datasets in both the populations. To differentiate the *hk* markers from male and female specific datasets, the AX prefix of these markers was replaced with HAX.
5. The homologous male and female linkage groups in the two populations were selected based on the common HAX markers.
6. Except two homologous pairs in ‘SB’ × ‘MK’ population and one in ‘D’ × ‘MK’ population, all homologous male and female linkage groups easily combined to form integrated maps.
7. To enable pairing of the unpaired homologus groups, the whole markers dataset of heterozygous SNPs (*hk*) (2539 SNPs from ‘D’ × ‘MK’ population and 1854 SNPs from ‘SB’ × ‘MK’ population) were again tested and markers with relaxed significance (*p*<0.005) were selected.
8. The additional HAX markers from step 7 were added to the previous 121 hk markers dataset, which facilitated pairing in all the homologous linkage groups.
9. The excessive HAX markers on certain homologous linkage groups were also thinned.
10. Finally 114 *hk* markers in ‘SB’ × ‘MK’ and 112 *hk* markers in ‘D’ × ‘MK’ populations were selected for pairing the homologous male and female linkage groups.
11. Of the selected *hk* markers, 91 were common between the two populations.
